# Pathological neural and immune synapses in glioblastoma progression: mechanisms and therapeutic opportunities

**DOI:** 10.3389/fncir.2026.1919817

**Published:** 2026-09-09

**Authors:** Ashwin Jainarayanan, Aishwarya Vedula, Neeraj Soni

**Affiliations:** 1 Department of Neurology and Neurological Sciences, Stanford Medicine, Stanford University, Stanford, CA, United States; 2 Kennedy Institute of Rheumatology, Nuffield Department of Orthopaedics, Rheumatology and Musculoskeletal Sciences (NDORMS), University of Oxford, Oxford, United Kingdom; 3 Department of Physiology, Anatomy and Genetics, University of Oxford, Oxford, United Kingdom; 4 Department of Pediatrics, Yale School of Medicine, Yale University, New Haven, CT, United States

**Keywords:** cancer neuroscience, glioblastoma, immunological synapses, neuroimmune interactions, neuron-glioma synapse, synaptic signaling

## Abstract

Glioblastoma (GBM), a high-grade glioma, is the most common primary brain tumor in adults, with a dismal prognosis and a very high recurrence rate. Major challenges to effective treatment arise from the tumor’s locally aggressive, diffusely infiltrative nature and extensive intratumoral heterogeneity. Recent advances in cancer neuroscience have uncovered bidirectional neuron-glioma interactions including the formation of functional neuron-glioma synapses (NGS). Neuronal activity drives tumor growth and its synaptic integration into neural circuits; tumor-induced circuit remodeling reciprocally influences neural activity. In parallel, emerging evidence implicates the immunological synapse- a dynamic interface between T cells and antigen-presenting cells analogous to its neural counterpart- as a key driver of GBM pathogenesis and progression. The tumor microenvironment (TME) in GBM is characterized by local immunosuppression, immune evasion, and resistance to immunotherapy. Notably, neural and immune synapses share fundamental architectural and functional principles–spatial confinement of signaling, receptor clustering and adhesion-mediated stabilization, directional information transfer, and use-dependent plasticity–and an expanding catalog of molecular hubs operates across both interfaces. In this context, glioblastoma can be reconceptualized as a disease of coupled neural and immune synaptic networks cooperating to drive tumor progression. Therapeutic strategies targeting these hubs may offer advantages over current approaches by simultaneously disrupting tumor-promoting neuronal input and enhancing antitumor immunity, providing a “double hit” with the potential to dismantle the network architecture sustaining tumor growth and resilience.

## 1 Introduction

Glioblastoma, a high-grade glioma and the most common primary malignant brain tumor in adults, is an aggressive cancer carrying a dismal prognosis (Louis et al., 2021; Ostrom et al., 2024). The 2021 WHO Classification of CNS Tumors restricted “glioblastoma” to IDH-wildtype disease, separating it from IDH-mutant astrocytoma (which now subsumes lesions previously called secondary glioblastoma) and from H3K27M-altered diffuse midline glioma (DMG), which is biologically and therapeutically distinct. This review focuses primarily on adult IDH-wildtype glioblastoma (hereafter referred to as “glioblastoma” or abbreviated as GBM) and pediatric DMG unless indicated otherwise. GBM is one of the most lethal human cancers, with median overall survival from diagnosis averaging 12–15 months and fewer than 7% of patients surviving 5 years (Stupp et al., 2017; Ostrom et al., 2020). The current standard of care–maximal safe resection followed by radiotherapy with concurrent and adjuvant temozolomide (Stupp et al., 2005; Weller et al., 2021; Kotecha et al., 2023) –has not been substantively improved for two decades, and recurrent disease remains essentially uniformly fatal (Wen and Kesari, 2008; Wirsching et al., 2021).

Two major GBM hallmarks underlie its intractability: its diffusely infiltrative nature (tumor cells typically extend centimeters beyond imaging-defined margins along white-matter tracts and perivascular spaces) (Claes et al., 2007; Cuddapah et al., 2014) and extensive intratumoral heterogeneity (ITH) (Sottoriva et al., 2013; Patel et al., 2014). Single-cell transcriptomic studies have revealed that glioma cells exist in a small number of cellular states that recapitulate developmental programs of the neural lineage–neural progenitor-like (NPC-like), oligodendrocyte progenitor-like (OPC-like), astrocyte-like (AC-like), and mesenchymal-like (MES-like)–and transition between these states with significant plasticity (Neftel et al., 2019; Eberhart and Bar, 2020; Wang et al., 2022; Lu et al., 2025). Deep and single-cell sequencing studies have also shed light on the much-debated cell of origin in GBM, implicating oligodendrocyte progenitor cells and their precursors, and subventricular zone (SVZ) neural stem cells that acquire oncogenic driver mutations, as the most likely candidates (Liu et al., 2011b; Galvao et al., 2014; Lindberg et al., 2014; Lee et al., 2018). These findings support the conceptual hallmark of oncogeny recapitulating and subverting ontogeny. Indeed, just as the proliferation, migration, and maturation of neural progenitors are governed by neuronal activity-dependent mechanisms and synaptic signals (Deisseroth et al., 2004; De Biase et al., 2010; Ohtaka-Maruyama et al., 2018), so too are their malignant counterparts. Over the last decade, neuronal activity has emerged as a major driver of GBM progression, invasion and therapeutic resistance, exerting its effects through secreted paracrine factors and direct synaptic input to the tumor. The discovery of bona fide neuron-glioblastoma synapses (NGS) has heralded the field of “cancer neuroscience,” presenting exciting new therapeutic avenues (Monje et al., 2020; Mancusi and Monje, 2023).

Another key determinant of GBM progression and clinical outcome is the tumor immune landscape. Despite frequent immune infiltration, the tumor microenvironment (TME) in GBM is characterized by profound local immunosuppression, immune evasion and resistance to immunotherapy (Quail and Joyce, 2017; Jackson et al., 2019; Friebel et al., 2020; Klemm et al., 2020; Sampson et al., 2020). Tumor-associated macrophages (TAMs), comprising both CNS-resident microglia and infiltrating monocyte-derived macrophages, constitute up to one-third of all cells in the tumor and dominate the immune compartment, while T cells are sparse, oligoclonal, and dysfunctional (Friebel et al., 2020; Klemm et al., 2020; Mathewson et al., 2021; Pombo Antunes et al., 2021). Both TAMs and myeloid-derived suppressor cells (MDSCs) in the glioblastoma TME exhibit an immunosuppressive phenotype, and support tumor growth and invasiveness. GBM cells downregulate the expression of key immune recognition molecules (such as MHC-I), upregulate immune checkpoint ligands facilitating immune escape (such as PD-L1) and release immunosuppressive cytokines (such as TGF-β, IL-10), thereby inhibiting effective T cell activation and promoting T cell exhaustion (Woroniecka et al., 2018; Himes et al., 2021). Single-agent immune checkpoint blockade has consistently failed in unselected glioblastoma populations (Reardon et al., 2020) and the disease has become a paradigm for the limits of contemporary immunotherapy. Recent spatial and multi-omic studies have begun to map cellular niches–perivascular, hypoxic, mesenchymal, and infiltrative–within which immune dysfunction is regionally organized (Hara et al., 2021; Greenwald et al., 2024).

The parallel emergence of glioma-neuron and glioma-immune interactions as drivers of tumor pathogenesis and progression have expanded its conceptualization from a cellular malignancy to a network disorder of dysregulated neuron-glioma-immune crosstalk. Importantly, both neural and immune cell signaling are mediated communication across a synapse- defined as “a stable adhesive junction between two cells in which information is relayed by directed secretion” (Dustin and Colman, 2002; Dustin, 2014). Neuron–neuron, neuron–glioblastoma, and immunological (formed between antigen-presenting-cell (APC)s and T-cells) synapses share the fundamental design and functional principles of organization (spatial confinement of signaling, receptor clustering and adhesion-mediated stabilization), communication (directional information transfer mediated by directed ligand secretion and binding to specific receptors), computation (intracellular signal integration and transformation), and adaptation (use-dependent plasticity). A growing catalog of molecules–including neuroligins, integrins, ephrins, semaphorins, BDNF–TrkB, glutamate receptors, CXCL12–CXCR4, PD-1–PD-L1–operates at both neural and immune synapses, providing a shared molecular vocabulary across systems. The emerging concept of the “neuroimmune connectome” recognizes that the nervous and immune systems constitute a single integrated network spanning cells, tissues and organs, whose fundamental signaling unit is the synapse (Filiano et al., 2016; Cserép et al., 2019; Wheeler and Quintana, 2025).

In this review, we synthesize the recent evidence for NGS and immunological synapse-mediated signaling in glioblastoma to argue that it can be understood as a network disorder of coupled neural and immune synaptic interfaces. We propose this neuron-tumor-immune connectome model of GBM as a conceptual framework to organize the experimental evidence and generate testable hypotheses, rather than a proven unifying mechanism. Within this framework, we posit that activity-dependent tumor growth and immune evasion are not parallel pathologies but two manifestations of a single underlying process: the systematic exploitation of synaptic communication by an infiltrating, plastic tumor cell network. We first review the molecular mechanisms of neuron–glioblastoma synapse formation and activity-dependent tumor progression, as well as the cellular and molecular landscape of the tumor immune microenvironment. We then discuss key molecular “hubs” that influence both neural and immune synaptic function, organized by their role in the four fundamental synaptic features outlined above. Finally, we summarize current therapeutic strategies targeting these hubs, stratify them by translational readiness and propose novel interventions that could serve as a “double hit” approach by simultaneously limiting activity-dependent tumor growth and enhancing antitumor immunity.

## 2 Neuron-glioblastoma synapses (NGS) in GBM circuit integration, activity-dependent tumor growth, and therapeutic resistance

### 2.1 Neuronal activity and neuron-glioblastoma synaptic interactions as drivers of tumor progression and resilience

The hypothesis that glioma cells respond to neuronal signals long predates direct demonstration. Scherer noted that infiltrating tumor cells preferentially associate with axons and white-matter tracts (“perineuronal satellitosis”) (Scherer, 1938), and Labrakakis showed that human glioma cells depolarize in response to glutamate, indicating functional neurotransmitter receptor expression (Labrakakis et al., 1998). More recent work has established that neuronal activity is not merely permissive but sufficient to drive tumor growth (Venkatesh et al., 2015, 2017). Indeed, even long-range (contrahemispheric) neural activity can drive glioma progression (Huang-Hobbs et al., 2023). Consistent with these experimental findings, clinical and imaging studies in GBM patients have found that tumor regions with high functional connectivity and elevated neuronal activity display increased expression of synaptogenic and invasion-associated genes, correlating with reduced survival (Krishna et al., 2023).

**FIGURE 1:**
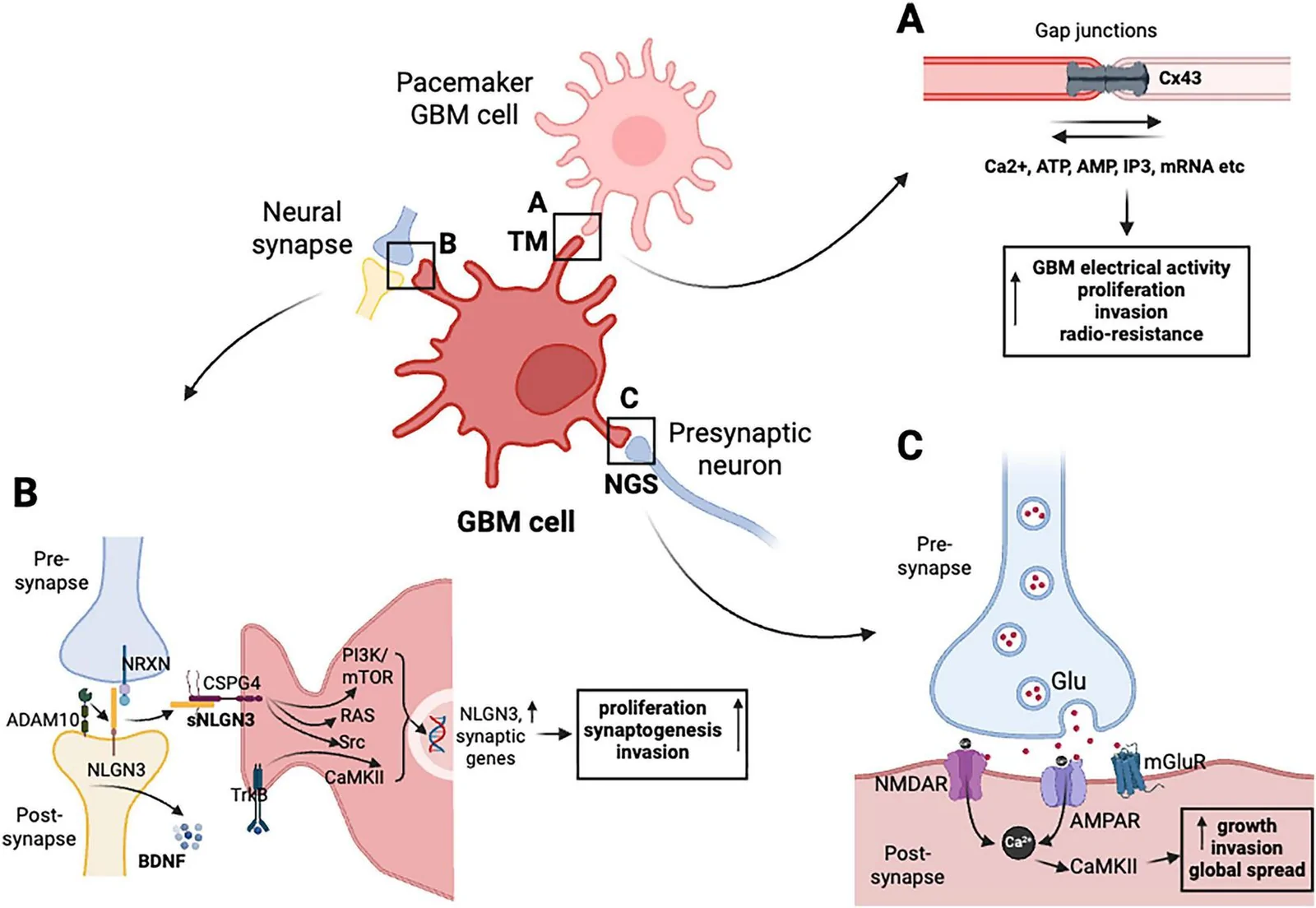
Neuronal activity-dependent mechanisms of glioblastoma (GBM) growth and integration into a functional network. **(A)** Tumor microtubes (TMs) couple GBM cells via gap junctions (containing Cx43) into an interconnected network, which is exploited by pacemaker cells within the tumor to synchronize tumor electrical activity. Exchange of various second messengers through the gap junctions allows for syncytial coupling of tumor cells for efficient information transfer. **(B)** Mechanisms by which peri-tumoral neuronal activity drives GBM growth. Neuronal activity increases ADAM10-mediated cleavage of NLGN3 into soluble NLGN3 (sNLGN3) and also results in increased BDNF secretion. These paracrine factors act on tumor cells to upregulate the expression of genes involved in synaptogenesis and cell proliferation, fueling further invasion and integration into the surrounding neural tissue. **(C)** Neuron-glioblastoma synapse (NGS) formed between presynaptic glutamatergic neuron and tumor microtube (TM) of GBM cell. Tumor cells express Ca^2+^-permeable AMPARs and other glutamate receptors, and Ca^2+^ signaling downstream of NGS results in increased tumor growth and invasion, facilitating global spread. Created in BioRender 2026.

Two parallel lines of investigation have identified the structural and molecular basis of neuron-glioblastoma cell communication and activity-dependent tumor progression. First, GBM cells extend ultra-long, dynamic membrane processes termed tumor microtubes (TMs) that infiltrate parenchyma along existing white-matter tracts and connect tumor cells to one another over distances of hundreds of micrometers (Osswald et al., 2015; Weil et al., 2017). TMs are actin- and tubulin-rich structures that contain mitochondria for local ATP production and morphologically resemble axonal growth cones (Osswald et al., 2015; Watson et al., 2023). A subset express the gap-junction protein connexin-43 (Cx43) at their tips, electrically and metabolically coupling glioma cells into a syncytial network in which “pacemaker” cells expressing the calcium-activated potassium channel KCa3.1 generate rhythmic Ca^2+^ transients that propagate across the tumor (Figure 1A; Osswald et al., 2015; Jung et al., 2017; Hausmann et al., 2022). This network underpins widespread tumor infiltration and resilience: ablation of TM-connected cells is followed by network repair from less-connected reservoir cells, and pharmacological or genetic disruption of TM formation sensitizes tumors to radiation and cytotoxic therapy (Weil et al., 2017; Venkataramani et al., 2022).

Second, several paracrine factors secreted as a result of neuronal activity have been identified to promote GBM proliferation, invasion and therapeutic resistance. The first to be molecularly characterized was neuroligin-3 (NLGN3), a postsynaptic adhesion molecule cleaved from neuronal membranes by the ADAM10 protease in an activity-dependent manner and released into the extracellular space (Venkatesh et al., 2015, 2017). Soluble NLGN3 binds to chondroitin sulfate proteoglycan-4 (CSPG4) on tumor cells–a canonical OPC marker overexpressed in GBM and pediatric DMG–and activates SRC kinase, the PI3K–mTOR axis, and downstream RAS signaling, driving robust proliferation (Figure 1B; Venkatesh et al., 2015, 2017; Gillespie et al., 2021, 2025). NLGN3 feed-forwardly induces its own expression and that of ADAM10, and also upregulates the expression of synapse-related genes in tumor cells. Genetic ablation of NLGN3 in the host or pharmacological inhibition of ADAM10 abolishes activity-dependent glioma growth in xenograft models, indicating that this signaling pathway is necessary and sufficient to drive tumor growth (Venkatesh et al., 2017). Contrahemispheric neuronal activity drives GBM growth through another activity-dependent paracrine factor, semaphorin 4f (Sema4f), which functions developmentally as an axon guidance molecule (Huang-Hobbs et al., 2023).

These streams of research converged on the discovery of bona fide glutamatergic neuron–glioblastoma synapses (NGS) (Figure 1C; Venkataramani et al., 2019; Venkatesh et al., 2019) between glioblastoma TMs and neurons in the infiltration zone, whose formation is regulated by NLGN3. Electron microscopy demonstrated classical synaptic vesicles in presynaptic neuronal boutons apposed to GBM cell membranes containing postsynaptic densities, and patch-clamp recordings reveal evoked excitatory postsynaptic currents in tumor cells with kinetics consistent with AMPA receptor activation. These currents are mediated by Ca^2+^-permeable AMPA receptors that lack edited GluA2 subunits, allowing direct Ca^2+^ entry on each synaptic event - a feature shared with neural progenitors and neurons during specific windows of synaptic plasticity (Venkataramani et al., 2019, 2022). Whole-brain colonization is driven preferentially by NPC-like and neuron-like glioma cells that are unconnected to the TM network but receive abundant synaptic input (Neftel et al., 2019; Venkataramani et al., 2022). Synaptic Ca^2+^ events in unconnected invasive cells are amplified intercellularly upon TM coupling, propagating through the network and driving downstream proliferative and invasive programs (Hausmann et al., 2022; Venkataramani et al., 2022). Furthermore, similar to their healthy counterparts, NGS exhibit adaptive plasticity regulated by brain-derived neurotrophic factor (BDNF) (Taylor et al., 2023). Activity-dependent BDNF–TrkB–CAMKII signaling increases NGS number and promotes AMPAR trafficking to the glioma cell membrane, resulting in increased synaptic strength.

Neuronal activity has thus emerged as a central driver of glioblastoma proliferation, invasion and therapeutic resistance through both paracrine (NLGN3, Sema4f, BDNF) signaling and electrochemical neuron-tumor synapses. It also represents a unique contribution to intratumoral heterogeneity: individual glioma cells within a tumor differ widely in their synaptic gene expression, amount and type of synaptic connectivity, and intrinsic electrical properties (Hausmann et al., 2022; Venkataramani et al., 2022). Recent studies integrating tumor cell characteristics, electrophysiology, functional connectivity and single-cell transcriptomics (Hausmann et al., 2022; Venkataramani et al., 2022; Taylor et al., 2023) have uncovered striking associations linking the three core axes shaping ITH: cellular transcriptional state, glioma cell-neuron communication, and malignant characteristics. MES-like and AC-like cells are enriched in the tumor core and display higher connectivity to surrounding cells. The glioma pacemaker cell marker KCa3.1 is enriched in MES-like cells. NEU- and NPC-like cells dominate the tumor’s infiltrative margin, receive neuronal synaptic input, are poorly connected to neighboring tumor and glial cells, and have higher motility, orchestrating brain-wide spread. NGS provide the activity-sensing input: glutamate released by presynaptic neurons drives AMPA-receptor–mediated depolarization and Ca^2+^ influx at postsynaptic densities on tumor cells. TMs, interconnected by connexin-43 gap junctions, then serve as the conductive substrate that propagates these signals as intercellular Ca^2+^ waves throughout the connected network, so that input received by a minority of synaptically-innervated cells is integrated into a network-wide proliferative and invasive response.

**FIGURE 2:**
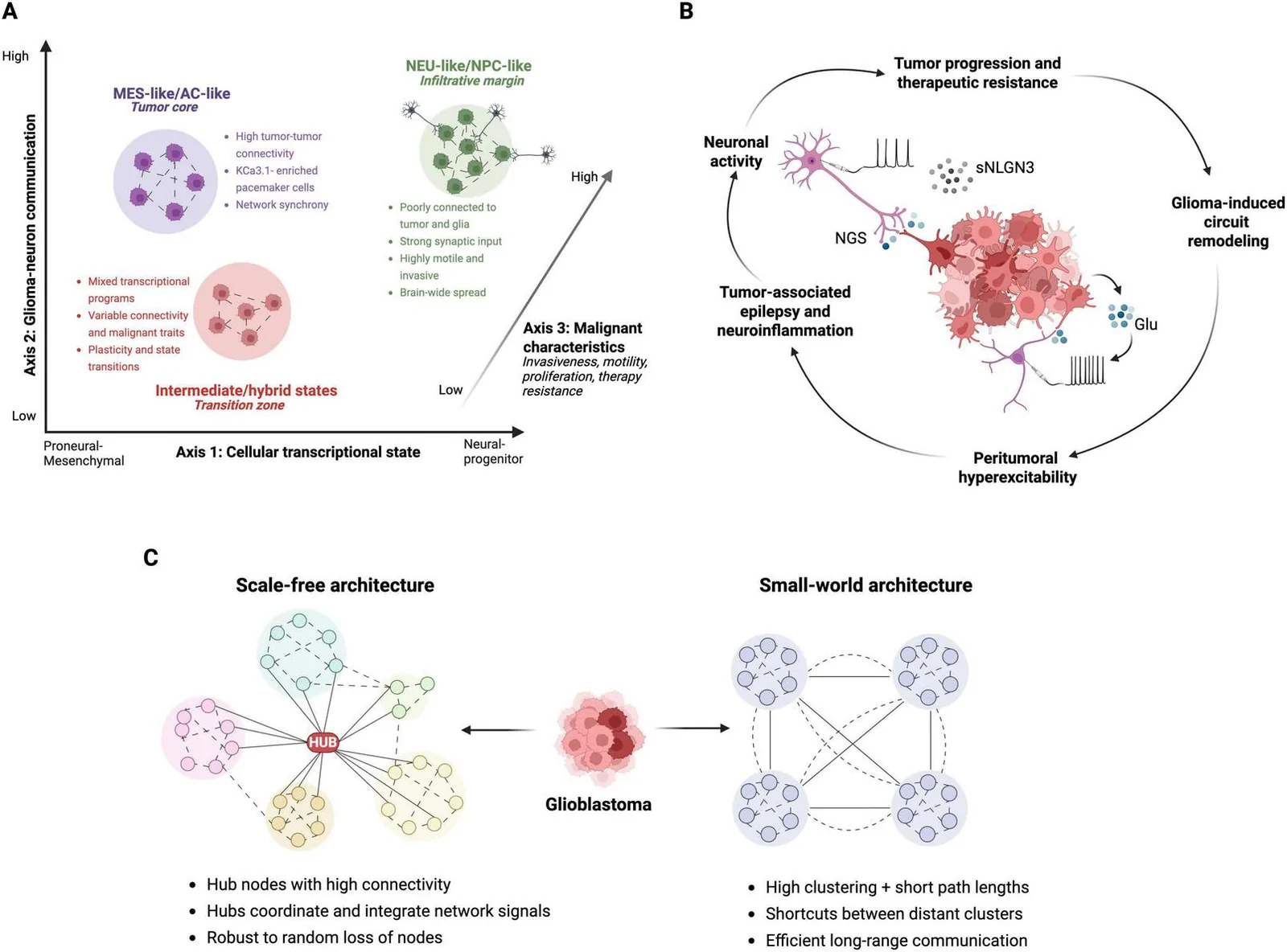
Glioblastoma-driven neural circuit remodeling and the architecture of the tumor cell network. **(A)** Intratumoral heterogeneity charted in a coupled state space defined by three interdependent axes: transcriptional state, neuron–glioma communication, and malignant phenotype. MES-/AC-like core cells are highly TM-connected, whereas NPC-/NEU-like margin cells receive synaptic input and orchestrate brain-wide spread. **(B)** The glioma–neural circuit remodeling “vicious cycle”: neuronal activity drives tumor growth, while the tumor reciprocally induces hyperexcitability through the cystine-glutamate antiporter (xCT) dysregulation and synaptic remodeling, producing tumor-associated epilepsy and feed-forward progression. **(C)** Small-world and scale-free architecture of the GBM cellular network. Created in BioRender 2026.

Neuronal activity-dependent mechanisms also directly influence cellular transcriptional state: synaptic activity and paracrine factors upregulate synaptic gene expression in glioma cells (Venkataramani et al., 2019; Venkatesh et al., 2019; Taylor et al., 2023), a transcriptional signature characteristic of neural, NPC- and OPC-like cells (Nomura et al., 2025). These findings could be reflected in distinct glioma cell clusters in the state space defined by the three interdependent ITH axes (Figure 2A), with neuronal activity acting both as an axis in its own right and as a driver of state transitions along the others. This conceptual framework enables the visualization of ITH in a higher-dimensional landscape where neuronal activity, transcriptional state, and malignant behavior are dynamic and tightly coupled, potentially uncovering novel cell states and enabling more refined subtyping of the GBM cellular landscape for targeted intervention.

An important caveat is that most mechanistic evidence for NGS derives from patient-derived xenografts in immunodeficient mice, which preserve neural circuitry but eliminate adaptive immunity, and from *in vitro* electrophysiology. Direct human validation, though more limited, is provided by intraoperative recordings in awake patients, in which glioma-infiltrated cortex shows functional connectivity that correlates with accelerated progression and impaired survival (Krishna et al., 2023). The extent to which each downstream molecular mechanism operates in immunocompetent human tumors remains to be fully established.

### 2.2 NGS-mediated neural circuit integration and remodeling by GBM cells

The bidirectionality of neuron–glioma interaction is now experimentally established. Brain-wide monosynaptic rabies-virus tracing in xenograft and genetically engineered models has shown that glioma cells receive both local and long-range synaptic input and integrate broadly into pre-existing circuits, with input patterns that closely mirror the native afferent landscape of the implanted region (Hsieh et al., 2024; Sun et al., 2025a; Tetzlaff et al., 2025). Reciprocally, GBM induces local and remote changes in neuronal structural and functional connectivity (Aabedi et al., 2022; Krishna et al., 2023). Glioma-innervating neurons themselves exhibit altered intrinsic and synaptic properties, indicating that the tumor reshapes the upstream network (Hsieh et al., 2024). Non-invasive transcranial magnetic stimulation enables clinically tractable mapping of these connectivity properties (Drexler et al., 2024).

Tumor-driven circuit remodeling has profound clinical consequences. Glioma cells secrete substantial amounts of glutamate via the cystine–glutamate antiporter xCT–a mechanism that simultaneously imports cystine for glutathione synthesis and exports glutamate at concentrations sufficient to depolarize and excitotoxically injure peritumoral neurons (Ye and Sontheimer, 1999; Buckingham et al., 2011). Concurrent upregulation of aquaporin-4 and downregulation of astrocytic Kir4.1 impair extracellular K^+^ buffering, depolarizing peritumoral neurons further (Binder et al., 2012; Madadi et al., 2021). The combined effect is hyperexcitability and tumor-associated epilepsy (TAE), which affects more than two-thirds of patients during their illness, is independently prognostic, and is mechanistically intertwined with tumor growth: neuronal activity drives proliferation, while tumor-derived glutamate drives activity (Köhling et al., 2006; Hills et al., 2022; Curry et al., 2023; Figure 2B). Recent work has identified IGSF3-mediated potassium dysregulation as a specific molecular driver of this epileptogenic loop (Curry et al., 2023).

Topologically, the GBM cellular network exhibits both small-world and scale-free organization similar to healthy neuronal networks (Figure 2C; Hausmann et al., 2022; Shyntar and Hillen, 2026). Small-world networks, characterized by high local clustering and short path lengths, allow rapid and efficient information flow throughout the network (Strogatz, 2001). Scale-free networks, defined by the presence of highly connected hub nodes, are resilient to random disruptions but vulnerable to hub node disruption. These topological properties allow for rapid signal integration and network resilience to focal disruption across the tumor. By integrating into highly connected nodes or hubs within existing neural networks, GBM cells can access disproportionate levels of activity-dependent signaling.

The integration of GBM cells into host neural circuits thus establishes a vicious cycle in which tumor cells both exploit and reshape neural circuitry to maintain continuous growth and progression, the interruption of which may be crucial to effective treatment.

We note, however, that the causal architecture of this cycle is only partially established. The human evidence is correlative: the degree of functional glioma–cortex connectivity is associated with faster progression and shorter survival, but association alone cannot establish direction (Krishna et al., 2023). Experimental interruption of neuronal activity slows tumor growth (Venkataramani et al., 2019; Venkatesh et al., 2019), supporting a causal contribution of activity itself. Direct evidence that activity-dependent circuit remodeling drives progression – rather than co-occurring with it, or arising as a consequence of tumor infiltration – is still lacking. Disentangling these possibilities, most plausibly through selective manipulation of remodeling in the absence of changes in bulk neuronal activity, remains a key experimental gap.

## 3 The glioblastoma immune environment and the role of immune synaptic signaling

### 3.1 The cellular landscape of the glioblastoma immune microenvironment

Single-cell transcriptomic and high-dimensional proteomic atlases have transformed understanding of glioblastoma immunity. The dominant immune compartment is myeloid: TAMs constitute 30%–50% of all viable cells in many tumors and include both CNS-resident microglia and infiltrating monocyte-derived macrophages, which can be distinguished by ontogeny and transcriptional signature (Müller et al., 2017; Friebel et al., 2020; Klemm et al., 2020; Pombo Antunes et al., 2021). Microglia-derived TAMs predominate in cortical and infiltrative regions, whereas monocyte-derived TAMs accumulate preferentially in perivascular and hypoxic/necrotic niches and are most strongly associated with immunosuppression and matrix remodeling (Pombo Antunes et al., 2021; Greenwald et al., 2024). The conventional M1/M2 dichotomy poorly captures this heterogeneity; current evidence supports a continuum of transcriptional states with overlapping pro- and anti-inflammatory features (Müller et al., 2017; Pombo Antunes et al., 2021). “Myeloid-derived suppressor cells,” originally described as a separate population, are increasingly viewed as immature monocyte-derived TAM intermediates; their precise classification remains debated.

Lymphoid infiltration is comparatively sparse and dysfunctional. CD4^+^ and CD8^+^ T cells in glioblastoma exhibit signatures of exhaustion–high PD-1, TIM-3, LAG-3, and TIGIT expression with low effector cytokine production and reduced clonal expansion (Friebel et al., 2020; Mathewson et al., 2021). Antigen-specific repertoires are narrow, with limited recognition of patient-specific neoantigens despite their presence (Mathewson et al., 2021). Regulatory T cells (Tregs) are enriched and contribute to suppression via IL-10, TGF-β, and CTLA-4-mediated antigen-presenting-cell modulation. A major upstream contributor to T cell paucity is bone marrow sequestration of naïve T cells via tumor-induced loss of surface S1P1, which traps cells in the marrow and prevents trafficking to the tumor (Chongsathidkiet et al., 2018). This non-synaptic mechanism constrains every immune-synapse-targeted strategy, because the synapse-forming partner is physically absent from the tumor.

Reactive astrocytes are increasingly recognized as active immunomodulators rather than passive stromal bystanders. Tumor-derived IL-11 reprograms astrocytes via STAT3 to express TRAIL, which induces apoptosis of CD4^+^ and CD8^+^ T cells in the tumor microenvironment. Disruption of the IL-11–STAT3–TRAIL axis enhances T cell and macrophage antitumor activity in preclinical models, and high IL-11 expression predicts poor survival in patients (Faust Akl et al., 2025). This circuit illustrates how glioblastoma “educates” stromal cells into an active immunosuppressive role and exemplifies a synapse-level mechanism–a TRAIL–DR4/5 contact functioning to actively eliminate antigen-specific lymphocytes.

Spatial profiling has begun to map this complexity onto tumor architecture. Greenwald and colleagues identified four major mesoscale niches: neuronal/infiltrative, mesenchymal/inflammatory, vascular, and hypoxic–each characterized by distinct combinations of tumor-cell states, immune populations, and stromal partners (Greenwald et al., 2024). NGS-rich infiltrative regions are enriched for NPC-like tumor cells and microglia-derived TAMs; mesenchymal niches are enriched for monocyte-derived TAMs, MES-like tumor cells, and exhausted T cells. This spatial organization is the natural substrate for evaluating neural–immune coupling *in vivo*.

### 3.2 Immunological synapse architecture and its disruption in glioma

The immunological synapse is the structured interface between a T cell and an antigen-presenting cell across which T cell receptor (TCR) engagement, costimulation, and cytotoxic effector function are coordinated (Grakoui et al., 1999; Dustin and Colman, 2002; Dustin, 2014). The mature synapse is organized into supramolecular activation clusters (SMACs): a central cluster (cSMAC) enriched in TCR and CD28, surrounded by a peripheral ring (pSMAC) of LFA-1 and ICAM-1, with a distal phosphatase-rich domain (dSMAC) (Dustin and Springer, 1989; Grakoui et al., 1999). Phosphatase exclusion from the cSMAC permits sustained kinase signaling, the microtubule-organizing center polarizes towards the synapse, and lytic granules release into the synaptic cleft for targeted cytotoxicity. Sustained Ca^2 +^ flux through ORAI/STIM channels drives nuclear translocation of NFAT and the transcriptional program of T cell activation. The immunological synapse is therefore both a signaling computer and a secretory aperture, integrating multiple TCR microcluster events into a coherent cellular decision. Multiple features of glioblastoma converge to disable this interface. Tumor cells frequently downregulate MHC class I and antigen-processing machinery, weakening TCR engagement (Berghoff et al., 2017). Costimulatory ligands CD80/CD86 are sparsely expressed on tumor cells and on intratumoral myeloid cells, while inhibitory ligands, particularly PD-L1, are abundant on tumor and myeloid surfaces. PD-1–PD-L1 engagement disrupts cSMAC organization, recruits the SHP-2 phosphatase to the synapse, and dephosphorylates proximal TCR signaling components (Yokosuka et al., 2012; Hui et al., 2017), establishing what may be regarded as a pathological inhibitory synapse. TIM-3, LAG-3, and TIGIT contribute additional inhibitory signaling at the same interface (Mathewson et al., 2021). D-2HG in IDH-mutant disease impairs the Ca^2+^–NFAT axis and alters synapse-associated metabolism (Bunse et al., 2018; Notarangelo et al., 2022). Tumor- and stroma-derived TGF-β and IL-10 reduce ICAM-1 clustering and impair LFA-1-mediated adhesion required for stable synapse formation. The cumulative effect is failure of synapse maturation, reduced cytotoxic granule polarization, and progression to terminal exhaustion or activation-induced cell death–the latter actively driven, in glioblastoma, by the astrocytic IL-11–STAT3–TRAIL axis (Faust Akl et al., 2025).

A distinct but related mechanism is trogocytosis: the synaptic transfer of plasma-membrane fragments from tumor cells to T cells across the synapse, which removes target antigens from tumor cells and induces fratricide and dysfunction in CAR T cells (Hamieh et al., 2019; Olson et al., 2022). In glioblastoma CAR T trials, antigen loss and limited persistence are major causes of failure, and trogocytosis at the immune synapse is increasingly recognized as a mechanistic contributor (Bagley et al., 2024; Choi et al., 2024). Trogocytosis exemplifies the principle that glioblastoma not only fails to assemble productive immune synapses but actively repurposes synaptic architecture against the immune system.

### 3.3 Molecular hubs of immunological synapse failure

Beyond classical checkpoint pathways, several signaling axes shape immune-synapse assembly and outcome in glioblastoma. Chemokine signaling positions immune cells within and around the tumor: CXCL12–CXCR4 - which is required for normal TCR-driven synapse formation in healthy T cells (Cascio et al., 2015) is repurposed in glioblastoma to recruit immunosuppressive TAMs and myeloid-derived suppressor-cell-like populations. Conversely, CXCL9/10/11–CXCR3 signaling, which mediates effector T cell trafficking in immune-responsive cancers, is muted in glioblastoma cores due to interferon-pathway suppression and stromal barriers (Greenwald et al., 2024). The CD47–SIRPα “don’t eat me” axis is exploited by tumor cells to avoid phagocytic clearance by TAMs, and CD47 blockade with magrolimab and related agents is in clinical evaluation in CNS tumors (Gholamin et al., 2017; Hutter et al., 2019). Adhesion-mediated trafficking through ICAM-1/VCAM-1 and integrin pathways shapes leukocyte retention and synapse stabilization at the tumor periphery and is altered by tumor- and TAM-derived inflammatory signals.

The IL-11–STAT3–TRAIL axis represents a particularly clean example of synapse-level immunosuppression: a tumor-derived cytokine reprograms a stromal cell into a T-cell-killing partner, in which the TRAIL–DR4/5 contact functions as a death synapse that actively eliminates antigen-specific lymphocytes (Faust Akl et al., 2025). STAT3 itself is a shared neural–immune hub, mediating both inflammatory plasticity in neurons and activation programs in immune cells (Yu et al., 2014), making it a strong candidate for combinatorial intervention. Galectin-9–TIM-3 signaling represents an additional inhibitory axis at the immune synapse with growing translational interest in CNS tumors. These molecular circuits do not operate in parallel but converge on a small number of synaptic outcomes: failure of synapse maturation, biased signaling toward inhibitory inputs, active induction of T cell death, and physical exclusion of effector cells from synaptically active tumor regions. Targeting these hubs provides the molecular basis for immune-synapse reprogramming.

## 4 Glioblastoma as a “synaptic” disorder: shared architectural and modulatory features across neural and immune synaptic interfaces

### 4.1 Scales for comparison of neural and immune synapses

A central premise of this review is that two distinct synapse types, neural and immune synapses, share design principles whose convergent disruption underlies glioblastoma progression. In the broadest conceptual sense, a synapse is an organized interface between cells designed for directed communication. By this definition, the GBM network includes several cell-cell contacts that exhibit synaptic features: neuron–neuron, neuron–tumor cell, tumor cell–APC, tumor cell–microglia, APC–T cell, and so on. In this section, we compare canonical neural (neuron–neuron) and immunological (APC–T cell) synapses, and extend the analytic framework to the GBM-specific neuron-glioblastoma synapses (NGS).

To carefully consider the similarities and differences across these synapse types, we must first precisely define the scales on which they are compared. Building on the multi-scale comparison proposed by Dustin and Colman (2002), neural, immune, and neuron–glioma synapses can be examined at conceptual, spatial, functional, and molecular levels.

**FIGURE 3:**
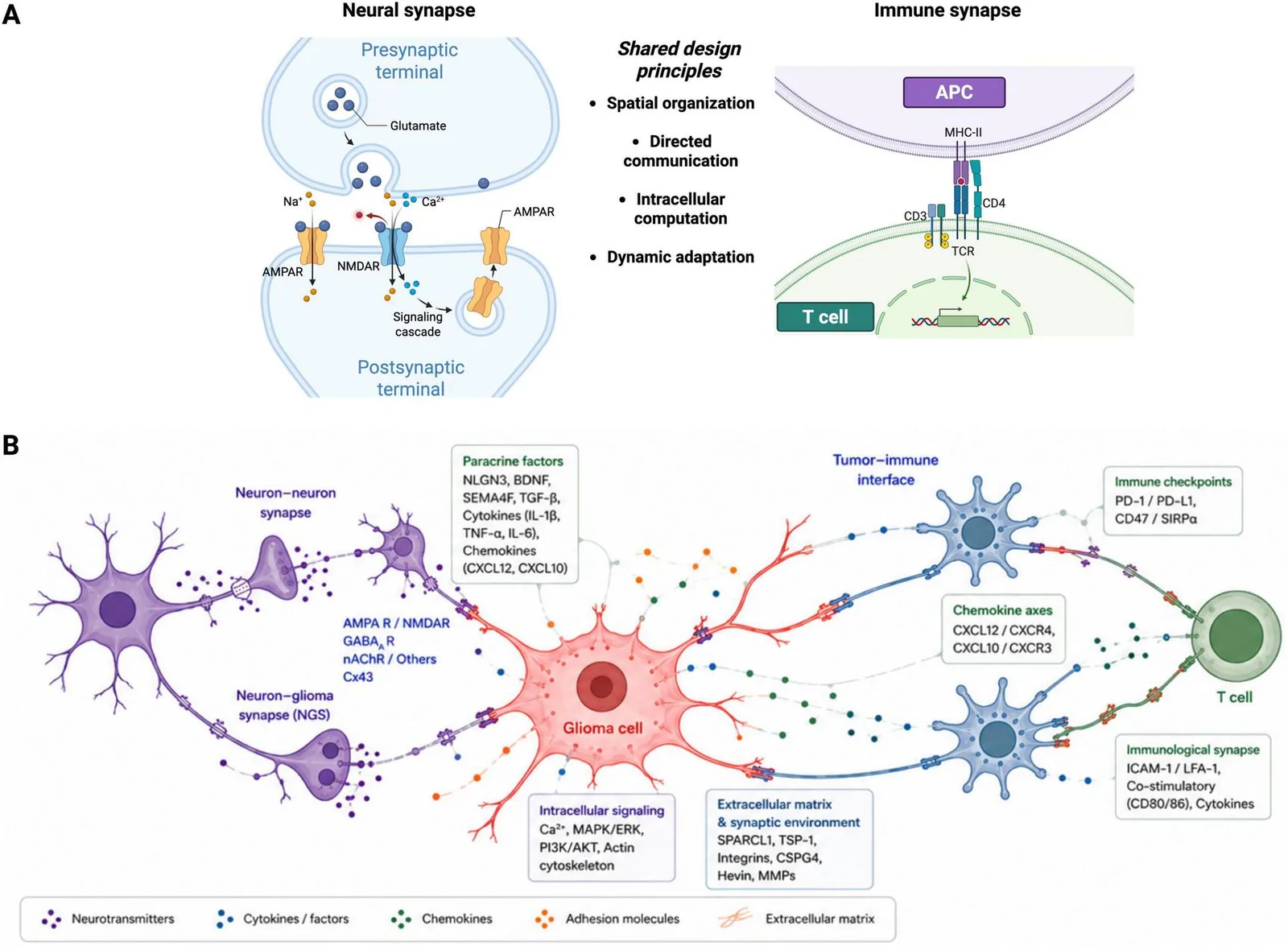
Shared design principles and molecular hubs across neural and immunological synapses in glioblastoma. **(A)** Neural and immunological synapses share fundamental design principles. **(B)** Molecular hubs subserving fundamental synaptic principles shared across neural and immune interfaces in glioblastoma. The glioma cell in panel **(B)** is intended to be representative of the potential synaptic interactions between neural, immune and tumor cells, and does not correspond to a particular cellular state. Created in BioRender 2026.

Conceptually, all three synapse types share the fundamental design principles of organization (spatial confinement of signaling, receptor clustering and adhesion-mediated stabilization), communication (directional information transfer with polarized organelles, specialized secretion machinery and receptors), computation (intracellular signal integration and transformation), and adaptation (use-dependent plasticity that strengthens or weakens the interface in response to repeated activation) (Figure 3A). The major points of conceptual difference between neural and immune synapses are their wiring properties and persistence. Neuronal synapses in the CNS are mostly hard-wired during development by specific molecular cues and can last years. This anatomic stability and spatial precision allows neural synapses to transmit information on a sub-millisecond timescale with high fidelity. In stark contrast to this, immunological synapses are transient, assembled and disassembled over minutes to hours as rapidly migrating T cells serially scan APCs, making many random contacts (transient immunological kinapses) till a match is found to form a synaptic contact (Friedl and Gunzer, 2001; Stoll et al., 2002; Miller et al., 2004; Dustin, 2008).

On the spatial scale, both neural and immunological synapses have a microdomain (<1 μm) structure comprising central active zones of exocytosis and endocytosis encircled by adhesion domains. However, they have fundamentally different macrostructure: while a single neuronal synapse is small (∼1 μm) and dedicated to one signaling channel, an immunological synapse is large (∼5–10 μm) and integrates dozens to hundreds of TCR microclusters, costimulatory clusters, and adhesion zones simultaneously (Krummel and Cahalan, 2010; Dustin and Groves, 2012; Yang and Annaert, 2021). Thus, on the functional scale, the immune synapse more closely resembles a postsynaptic dendrite, or a small dendritic tree that integrates multiple inputs, than a single neural synapse (Dustin, 2014). An additional difference on the spatial scale is synapse position in relation to the cell’s nucleus. Neural synapses in the CNS are typically “action at a distance” junctions, and may be situated from a few microns to several cm away from the nucleus (Deisseroth et al., 2003; Adams and Dudek, 2005; Spruston, 2008; West and Greenberg, 2011; Stuart and Spruston, 2015). In contrast, the distance between a typical immune synaptic interface and the T cell nucleus averages a few micrometers (Stinchcombe et al., 2006; Dustin, 2014; Asano et al., 2025). This difference, too, has a functional consequence: immune synapses are often tightly coupled to rapid transcriptional reprogramming, whereas neural synapses frequently rely on local signaling and long-range communication with the soma before transcriptional responses are engaged.

Zooming in further, on the molecular scale, neural and immune synapses are characterized by distinct cytoskeletal bridging proteins and receptor expression profiles that drive signaling. However, a wealth of recent evidence indicates extensive bidirectional crosstalk between the nervous and immune systems mediated by proteins expressed in both (Boulanger, 2009; Kioussis and Pachnis, 2009).

Taken together, these observations suggest that neural and immunological synapses occupy different positions along a continuum of organizational and signaling strategies, and do not operate in isolation from each other. Across these conceptual, spatial, functional and molecular scales, the neuron-glioblastoma synapse (NGS) occupies a unique intermediate position between classic neural and immunological synapses. Conceptually, though NGS formation hijacks several of the molecular cues driving neural synapse formation, it is more indiscriminate and structurally plastic (Venkataramani et al., 2022; Taylor et al., 2023), enabling widespread tumor-neuron communication to fuel progression. Structurally, NGS closely resemble canonical neural synapses, with presynaptic vesicle release apposed to a postsynaptic density and AMPA receptor activation. Functionally, however, NGS are embedded in the broader TM-coupled tumor network, such that single-synapse Ca^2+^ events propagate intercellularly to activate transcriptional programs across hundreds of cells - an integrative function more reminiscent of an immune synapse and a neural dendritic tree than of a single neural synapse. Finally, several molecular hubs shared across neural and immune synapses also govern signaling at NGS (Table 1, section “4.3 Shared neural/immune synaptic hubs with key roles in GBM pathophysiology”), integrating neurons, tumor cells and immune cells into a single structural and functional network (Figure 3B).

**TABLE 1:** Summary of shared synaptic hubs linking neural and immune signaling in glioblastoma.

Table A. Architectural synaptic hubs (spatial organizers).				
**Synaptic hub**	**Neural synaptic/signaling role**	**Immune synaptic/signaling role**	**Role in GBM pathophysiology**	**Evidence/clinical status**
ICAM-1–LFA-1 (α_L_β_2_ integrin)	Injury-induced neuroinflammatory signaling; microglia-mediated immune surveillance and adhesion	Canonical adhesion pair stabilizing immunological synapse (pSMAC); T cell trafficking and activation	ICAM-1 overexpressed in GBM; perivascular invasion; microglial recruitment	Preclinical/early translational
αvβ3 integrin	Postsynaptic adhesion receptor regulating AMPAR surface delivery and stabilization; long-term potentiation	pSMAC protein regulating APC-T cell interaction; TGF-β activation	Expression correlates with tumor grade; perivascular invasion; senescence escape via αvβ3/PAK4 signaling	Clinical/phase III (cilengitide)
Connexin-43 (Cx43)	Gap junction coupling; astrocyte-neuron interactions	pSMAC protein; bidirectional transfer of second messengers e.g.: ATP, cAMP, IP3, Ca2+	Couples GBM cells into a functional network; regulates tumor invasion and therapy resistance	Clinical/early translational
Ephrin–Eph receptors	Axon guidance and synapse formation; dendritic spine morphogenesis; long-term potentiation	Regulates immune cell positioning through chemotaxis; modulation of T cell activation	GBM invasion, angiogenesis and therapy resistance; suppression of tumor cell apoptosis	Clinical/early translational
LAR-RPTPs (PTPσ/LAR/PTPδ)	Master organizers of synapse assembly and trans-synaptic adhesion through Slitrks, TrkC, SALMs	Inhibitory signaling checkpoints in T cells and pDCs; modulate cell migration; limited direct immune synapse evidence	RPTP tumor suppressor downregulated in GBM	Emerging
Neuroligin–neurexin	Canonical trans-synaptic adhesion complex driving synapse formation and specification	Emerging roles in immune cell communication and leukocyte-stromal interactions	NLGN3–CSPG4 mediates NGS formation, activity-dependent tumor growth and therapy resistance	Preclinical
Semaphorin–plexin	Axon guidance, synapse formation and structural plasticity	Immune suppression; T-cell exclusion	Sema4f-mediated contra-hemispheric tumor spread	Emerging
SPARCL1 (hevin)	Synaptogenic protein regulating excitatory synapses	ECM modulation affecting immune infiltration	NGS formation, tumor infiltration and angiogenesis	Emerging
Thrombospondin-1 (TSP-1)	Synaptogenesis and neuron–tumor coupling; enriched at tumor–brain interface	Activates TGF-β; regulates macrophage polarization	NGS formation, TM formation, invasion, regional immunosuppression	Clinical
Table B. Directed signaling hubs. These are subgrouped by their canonical/dominant role into neurotransmitters, chemokines, trophic factors and immune recognition signals.				
Synaptic hub	Neural synaptic/signaling role	Immune synaptic/signaling role	Role in GBM pathophysiology	Evidence/clinical status
Neurotransmitters				
Glutamate (AMPAR/NMDAR)	Drives neuron–glioma synaptic signaling; promotes proliferation	Modulates T-cell activation and myeloid-cell function	Major driver of activity-dependent tumor growth and invasion	Clinical/phase II (perampanel/NOA-30)
GABA	Regulates inhibitory tone within tumor-associated circuits	Suppresses T-cell activation and inflammatory responses	GABA polarity reversed in GBM; disinhibition contributes to TAE	Preclinical
Acetylcholine	Modulates neural circuit activity	Regulates immune responses (cholinergic anti-inflammatory axis)	CHRM3 upregulated by GBM; ACh-CHRM3 signaling drives tumor growth and therapy resistance	Emerging
Adenosine	Regulates neural activity	Strong immunosuppressive signaling (A2A receptor)	Promotes immune evasion in the TME	Clinical/early translational
ATP	Activity-dependent signaling; excitotoxicity	Pro-inflammatory danger signal (P2X/P2Y receptors)	GBM cells secrete ATP; couples neuronal activity to inflammatory signaling in the TME	Preclinical
Dopamine	Neuromodulates basal ganglia circuitry, synaptic plasticity and rewards-related signaling	Regulates T-cell activation, macrophage polarization and cytokine secretion through dopamine receptors	Signaling through DRD2 promotes tumor progression in H3K27M-mutant DMG	Clinical/approved (dordaviprone) for H3K27M DMG, early translational for GBM
Norepinephrine	Regulates arousal-dependent circuit activity and synaptic gain	Modulates immune cell trafficking and activation via adrenergic receptors	Can promote (through Twist1) or inhibit (through decreased MMP-11) tumor cell migration and invasion	Emerging
Chemokines				
CXCL12–CXCR4	Neuronal migration and guidance	Directs T-cell, monocyte and microglial trafficking; regulates immune-cell retention and positioning	CXCR4 overexpressed by GBM cells; promotes cell motility, proliferation, invasion and therapy resistance	Clinical/phase I (plerixafor combinations)
CXCL10–CXCR3	Mediates neuroinflammatory signaling and neuron–glia communication during injury and disease	Recruits activated T cells and NK cells; regulates immune-cell localization and activation	Dual role; CXCL10/CXCR3 upregulated in GBM cells; autocrine loop promotes tumor survival but paracrine signaling can be leveraged to recruit antitumor CD8+ T cells to the tumor	Emerging
Trophic factors				
sNLGN3–CSPG4	Activity-dependent glioma growth	Limited evidence	Central mediator of neural activity-dependent tumor growth	Preclinical
BDNF–TrkB	Activity-dependent neurotrophin regulating synaptic plasticity, dendritic remodeling and neuronal survival	Modulates immune-cell survival, activation and antigen-presenting capacity	Key mediator of neural activity-dependent tumor growth and plasticity at neuron-GBM synapses	Emerging
Immune recognition signals				
MHC-I–TCR (PirB/LILRB2)	Activity-dependent synaptic refinement, pruning and neuronal plasticity	Core antigen-recognition machinery of the immune synapse; initiates T-cell activation and adaptive immune response	MHC-I downregulated by GBM cells to facilitate immune escape	Emerging/early translational
CD47–SIRPα	Regulates microglia-mediated synaptic pruning; protects neuronal synapses from inappropriate phagocytosis	Canonical “don’t eat me” checkpoint suppressing macrophage and microglial phagocytosis through SIRPα	Integrates neuronal synapse preservation with tumor immune evasion	Clinical/phase I [anti-CD47: magrolimab (PNOC025)]
Table C. Signal integration synaptic hubs.				
Synaptic hub	Neural synaptic role	Immune synaptic role	Role in GBM pathophysiology	Evidence/clinical status
Ca^2+^–CaMKII/CaMKK2/Calcineurin	Couples synaptic activity to calcium-dependent signaling, plasticity and activity-dependent gene expression	Couples TCR signaling to NFAT activation and immune-cell activation	Integrates synaptic activity into tumor growth, transcriptional reprogramming and immune responses	Preclinical
MAPK/ERK	Mediates activity-dependent synaptic signaling, plasticity and neuronal survival	Integrates TCR and cytokine signaling to regulate activation, proliferation and differentiation	Promotes tumor proliferation, invasion and adaptive responses to neural activity	Clinical/phase II (MEK inhibitors trametinib/selumetinib in MAPK-altered gliomas)
PI3K/AKT/mTOR	Couples synaptic receptor activation to protein synthesis, metabolism and neuronal survival	Regulates immune-cell metabolism, activation and effector differentiation	Coordinates tumor growth, metabolic adaptation and immunosuppressive signaling	Clinical/phase II (buparlisib, paxalisib)
STAT3	Integrates synaptic activity and cytokine signaling to regulate neuronal and glial transcriptional programs	Central regulator of macrophage polarization and T-cell dysfunction	Major convergence node linking neural activity to tumor growth and immune suppression	Clinical/phase I (WP1066)
Table D. Synaptic hubs involved in dynamic plasticity.				
Synaptic hub	Neural synaptic/signaling role	Immune synaptic/signaling role	Role in GBM pathophysiology	Evidence/clinical status
TNF-α	Regulates homeostatic synaptic scaling, AMPAR trafficking and activity-dependent plasticity	Modulates immune synapse strength, T-cell activation and inflammatory polarization	Couples chronic inflammation to adaptive remodeling of neural and immune signaling, promoting tumor progression and immune dysfunction	Preclinical
IL-1β/IL-1R	Regulates synaptic remodeling, excitability and activity-dependent plasticity	Controls innate immune activation, antigen presentation and T-cell priming	Drives neuroinflammation, circuit remodeling and establishment of an immunosuppressive tumor microenvironment	Preclinical
PD-1/PD-L1	Suppresses neuronal excitability and synaptic plasticity	Canonical immune checkpoint regulating immune synapse signaling, T-cell activation and exhaustion	Facilitates tumor immune evasion	Clinical/phase III

Synaptic hubs are grouped according to the synaptic function they most strongly exemplify: tight spatial organization (A), directed signaling (B), intracellular signal integration (C), and dynamic plasticity (D). Hubs were selected based on evidence linking them to both neural and immune synaptic signaling and their relevance to glioblastoma biology. Contributions to the two synapse types are not necessarily equivalent; where direct evidence is limited, physiological synaptic roles or plausible GBM mechanisms are indicated. Evidence categories are descriptive rather than quantitative. “Emerging”– initial evidence from GBM studies (typically *in vitro* and/or exploratory *in vivo*); “Preclinical”– substantial functional evidence from multiple *in vitro* and *in vivo* GBM models; “Clinical”– substantial evidence from human GBM specimens and/or clinical studies. Clinical status summarizes the highest stage of therapeutic development in GBM. “Early translational”– human therapeutic rationale established (e.g., biomarker studies, target validation in patient tissue, first-in-human studies in other diseases or cancers) but no completed GBM clinical trial; “Phase I–III”– phase of human GBM clinical trial ongoing/completed; “Approved”–therapeutic approved for clinical use in GBM. Representative primary studies supporting the physiological synaptic roles of each hub and their dysregulation in glioblastoma are discussed and cited in the main text.

### 4.2 Molecular crosstalk across neural, immune and neuron-glioblastoma synapses

Decades of work on neuroimmune interaction document extensive bidirectional crosstalk between the nervous and immune systems (Filiano et al., 2016; Wheeler and Quintana, 2025). Most of the experimental evidence for neural/immune crosstalk centers on their overlapping expression of directed signaling systems. Neurons express receptors for inflammatory cytokines including TNF-α, IL-1β, IL-6, and type I interferons, and inflammatory signaling modifies neuronal excitability, synaptic plasticity, and circuit dynamics. Microglia and astrocytes participate directly in synaptic pruning during development and in neurodegeneration via complement and TREM2-related pathways (Stevens et al., 2007). Cserép et al. (2019) identified somatic purinergic junctions between microglia and neuronal cell bodies that exhibit specialized molecular architecture–mitochondrial accumulation, vesicle clusters, scaffolding by Kv2.1–and meet the operational criteria of a neuroimmune synapse. These junctions are themselves remodeled in pathological states, providing a direct demonstration that neuronal and microglial cells engage in structured, contact-dependent signaling with synaptic organization.

Conversely, immune cells express receptors for classical neurotransmitters. T cells, B cells, and myeloid cells express functional glutamate receptors (both ionotropic and metabotropic), GABA receptors, dopamine and acetylcholine receptors, and adrenergic receptors, and their activation modulates effector function, migration, and differentiation (Ganor and Levite, 2014; Wheeler and Quintana, 2025). Glutamate signaling lowers T cell activation thresholds at moderate concentrations but induces dysfunction at the high concentrations characteristic of the glioblastoma microenvironment. Adenosine and GABA contribute to immunosuppression, while β-adrenergic signaling broadly inhibits effector function (Magnon et al., 2013; Allard et al., 2020). In the CNS, peripheral T cells have been shown to influence behavior and cognition through cytokine release into meningeal spaces (Filiano et al., 2016), and meningeal lymphatic drainage to deep cervical lymph nodes is a critical determinant of CNS antitumor immunity (Louveau et al., 2015; Hu et al., 2020).

Within glioblastoma, the integration of these two streams is not yet directly demonstrated at synaptic resolution, but multiple lines of indirect evidence support functional coupling. Spatial transcriptomics shows colocalization of synaptically active tumor regions with specific TAM transcriptional states (Greenwald et al., 2024). BDNF, glutamate, NLGN3, and acetylcholine–all upregulated by neuronal activity in the tumor microenvironment have known immunomodulatory effects in other tissues. Acute and chronic stress, via β-adrenergic signaling, alter both peripheral immunity and central neuronal activity, and activity-dependent immune modulation has been documented in non-CNS cancers (Magnon et al., 2013; Mancusi and Monje, 2023).

### 4.3 Shared neural/immune synaptic hubs with key roles in GBM pathophysiology

We define a GBM-relevant “synaptic hub” operationally as a molecular system that (i) has a well-established functional role at canonical neural and/or immunological synapses, (ii) participates in both neural and immune cell signaling, and (iii) has a demonstrated or plausible role in GBM pathophysiology. This broad definition recognizes that the contribution of a given hub to the two synapse types is not necessarily equivalent: some have well-established fundamental roles in either neural or immune synaptic signaling, and more indirect, emerging or context-dependent roles in the other. Synaptic hubs can be grouped according to their best-characterized functional role, corresponding to the four fundamental synaptic design principles detailed previously: spatial organization (architecture; Table 1A), directed signaling (communication; Table 1B), intracellular signal integration (computation; Table 1C) and dynamic plasticity (adaptation; Table 1D).

Architectural hubs (Table 1A) serve as spatial organizers in neural and immunological synapses. Recent studies have shown that canonical synaptic organizers, such as the immune adhesion molecule ICAM-1 and the neural adhesion receptor αvβ3 integrin, frequently function across both systems and play key roles in GBM pathogenesis and progression. ICAM-1, which interacts with the integrin LFA-1 to drive immunological synapse formation and T cell trafficking, is upregulated on glial cells and neurons in response to injury and inflammatory cytokines, mediating leukocyte and microglial recruitment and neuroinflammatory pain signaling (Birdsall, 1991; Lutton et al., 2017; Chaudhari et al., 2025). ICAM-1 is frequently overexpressed in GBM (Piao et al., 2017; Guo et al., 2025), controlling tumor stemness and proliferation through Wnt/β-catenin signaling. ICAM-1 regulates PD-L1 expression through β-catenin signaling, promoting immune evasion in the TME, and ICAM-1 inhibition synergizes with anti-PD-1 therapy in immunocompetent GBM xenograft models (Guo et al., 2025). GBM cells also secrete soluble ICAM-1 via FASN signaling/STAT-1 signaling, which promotes a mesenchymal shift of tumor cells, TAM infiltration, microglial recruitment and radio-resistance (Yoo et al., 2022; Zhang et al., 2025).

In neural synapses, αvβ3 integrin regulates postsynaptic AMPAR stabilization and long-term potentiation; it also contributes to immune synapse formation (particularly in T_*H*_2 cells) and TGF-β activation. In GBM, αvβ3 expression correlates with tumor grade, promotes invasion and enables senescence escape through αvβ3–PAK4 signaling (Franovic et al., 2015). Similarly, the neuroligin–neurexin adhesion complex canonically specifies neuronal synapses (Chanda et al., 2017; Südhof, 2017), but in GBM, neuronal activity-dependent shedding of NLGN3 (sNLGN3) and signaling through CSPG4 has been co-opted to drive NGS formation, tumor growth and therapeutic resistance (Section “2.1 Neuronal activity and neuron-glioblastoma synaptic interactions as drivers of tumor progression and resilience”). Connexin-43 (Cx43)- containing gap junctions are a hallmark of electrical synapses in the CNS; Cx43 also contributes to signaling between immune cells (Mendoza-Naranjo et al., 2011) and plays a central role in GBM pathogenesis and progression by electrically and metabolically coupling tumor microtubes into a functional multicellular network that promotes invasion and therapy resistance (Osswald et al., 2015).

Signaling axes that serve as developmental guidance systems for neural synapse formation have also been shown to play important roles in immune cell signaling, and repurposed in GBM to drive tumor growth and invasion. Ephrin–Eph signaling regulates axon guidance, dendritic spine morphogenesis and immune-cell positioning, while promoting glioblastoma invasion, angiogenesis, apoptosis resistance and therapeutic escape (Day et al., 2013; Ribeiro et al., 2025). Semaphorin–plexin signaling likewise controls structural plasticity and immune-cell migration, and Sema4f is a key driver of activity-dependent, contralateral GBM spread (Yoshida, 2012; Huang-Hobbs et al., 2023). Extracellular matrix organizers such as SPARCL1 (Hevin) and thrombospondin-1 (TSP-1) orchestrate excitatory synaptogenesis in the healthy CNS (Christopherson et al., 2005; Eroglu et al., 2009; Gan and Südhof, 2020), and also play key roles in regulating immune cell infiltration and macrophage polarization (Martin-Manso et al., 2008; Kaur and Roberts, 2024; Zhao et al., 2024; Draganić et al., 2026). SPARCL1 has recently been implicated in neuron–glioma synapse formation, tumor infiltration and angiogenesis (Li et al., 2025; Allgood et al., 2026). TSP-1 is heavily upregulated by TGF-β signaling in GBM, and interacts with CD47 and CD36 to promote tumor cell proliferation, tumor microtube and NGS formation, and therapeutic resistance (Daubon et al., 2019; Joseph et al., 2022; Nejo et al., 2025). Interestingly, richly connected GBM regions exhibit TSP-1 upregulation and regional immunosuppression, and both TSP-1 knockout and glutamatergic inhibition enhance antitumor immunity (Nejo et al., 2025), illustrating the multiple neural and immune roles of this hub. Gabapentinoids, which are pharmacological inhibitors of TSP-1, confer a survival benefit in GBM (Bernstock et al., 2025).

Several architectural hubs remain less extensively characterized in GBM but highlight important emerging areas. LAR-family receptor protein tyrosine phosphatases (LAR-RPTPs) are master organizers of trans-synaptic adhesion through interactions with Slitrks, TrkC and SALMs (Takahashi and Craig, 2013; Sakamoto et al., 2021), while also modulating immune-cell migration and inhibitory signaling, although direct immune synapse evidence remains limited. Recurrent dysregulation of LAR-family phosphatases in GBM suggests they may represent emerging neuroimmune organizers (Veeriah et al., 2009; Ortiz et al., 2014).

A growing number of hubs subserving directed signaling across the nervous and immune systems (section “4.2 Molecular crosstalk across neural, immune and neuron-glioblastoma synapses”)–including soluble neurotransmitters, chemokines, trophic factors and membrane-bound recognition signals–operate at neural and/or immune synapses, and play key roles in GBM pathology (Table 1B). Following the discovery of glutamatergic neuron-glioma synapses and their pivotal role in tumor progression, glutamate remains the best-characterized neurotransmitter driving neuron–glioma synaptic interactions. Beyond AMPA receptors, NMDA, kainate, and metabotropic glutamate receptors - particularly mGluR3, enriched in proneural glioma stem cells- contribute to growth and chemoresistance (Ciceroni et al., 2013; Wirsching et al., 2021).

Recent studies have characterized neuron-glioblastoma synapses employing other neurotransmitters, such as gamma-amino butyric acid (GABA) (Barron et al., 2025) and acetylcholine (ACh) (Sun et al., 2025b). Compromised GABA-mediated inhibitory transmission is a key feature in GBM and contributes to tumor progression and TAE (Campbell et al., 2015; MacKenzie et al., 2016; Nissen et al., 2025). Glioblastoma and DMG overexpress the chloride importer NKCC1 and downregulate the chloride extruder KCC2, reversing the chloride gradient such that GABA-A-receptor activation is depolarizing in tumor cells (Campbell et al., 2015; MacKenzie et al., 2016; Barron et al., 2025). This recapitulates the developmental polarity of GABA, which is excitatory in immature neural progenitors and shifts to inhibitory upon KCC2 upregulation during postnatal maturation (Ben-Ari, 2002). In mouse xenograft models of GBM, depolarizing GABA signaling promotes proliferation and contributes to peritumoral hyperexcitability (Barron et al., 2025). Acetylcholine signaling also enhances tumor proliferation and drives tumor cell motility and invasion. Brain-wide rabies-virus retrograde tracing studies have revealed that GBM cells receive both local and long-range cholinergic input, and that activation of the muscarinic receptor CHRM3, overexpressed on tumor cells, transcriptionally reprograms tumors toward NPC- and OPC-like states with enhanced motility (Sun et al., 2025a; Tetzlaff et al., 2025). This is reminiscent of the role of cholinergic signaling in regulating NSC proliferation in the developing subventricular zone (Paez-Gonzalez et al., 2014).

Several other neurotransmitters play significant roles in glioblastoma pathogenesis and progression, although direct experimental evidence for NGS employing these signaling systems remains limited. The dopamine D2 receptor (DRD2) is upregulated in GBM and DMG and converges on EGFR–MAPK signaling (Li et al., 2014). Purinergic signaling supports both metabolic and immunosuppressive functions: astrocyte-derived ATP fuels invasive-edge tumor proliferation (Yang et al., 2025), while tumor-expressed CD39 and CD73 hydrolyze ATP to adenosine, which signals through A2A receptors to suppress effector T cells (Allard et al., 2020). Other neuromodulatory systems including norepinephrine, serotonin, histamine, and endocannabinoids have also been implicated in glioma progression, although their roles are less well defined and often context-dependent (Huang et al., 2022; Wang et al., 2025; Tralongo et al., 2026).

Chemokine axes with roles across neural and immune systems include CXCL12–CXCR4, originally characterized as a regulator of leukocyte trafficking but also essential for neural progenitor migration, axon guidance and cortical development (Li and Ransohoff, 2008), which is markedly upregulated in GBM, where it promotes glioma stem cell (GSC) maintenance, invasion, angiogenesis and therapeutic resistance (Mercurio et al., 2016) while contributing to immune cell recruitment and exclusion (Rubin et al., 2003; Ehtesham et al., 2006; do Carmo et al., 2010; Wu et al., 2022; Makranz et al., 2026). Combination anti-CXCR4 and anti-PD-1therapy provides a survival benefit in murine glioma models by enhancing antitumor immunity, compared to monotherapy (Wu et al., 2019). Similarly, CXCL10–CXCR3 signaling contributes to neuroinflammatory signaling and neuronal injury responses while regulating activated T-cell recruitment. In GBM, dysregulated CXCL10 signaling shapes inflammatory cell infiltration and influences responses to immunotherapy (Liu et al., 2011a; Madkhali et al., 2025; Zhang et al., 2026).

Activity-dependent trophic factors further reinforce tumor adaptation. BDNF–TrkB signaling supports neuronal plasticity, tumor progression and emerging immunomodulatory functions (Park and Poo, 2013; Sochal et al., 2022). Finally, membrane-bound recognition systems including MHC-I–TCR and CD47–SIRPα illustrate that contact-dependent signaling is also shared across neural and immune interfaces, linking neuronal synapse regulation with tumor immune evasion (Oldenborg et al., 2000; Shatz, 2009; Chao et al., 2012; Hutter et al., 2019; Whipple et al., 2025).

Neural and immune synaptic signaling also share a number of intracellular signal integration hubs that perform the computational step of converting synaptic inputs into coordinated cellular responses, and are dysregulated in GBM (Table 1C). Calcium signaling, acting through CaMKII, CaMKK2 and calcineurin, couples neuronal activity to long-term plasticity while simultaneously serving as the principal second messenger downstream of T-cell receptor activation (Hogan et al., 2003; Wayman et al., 2008; Lisman et al., 2012). Neuronal CaMKK2 promotes immunosuppression and resistance to checkpoint blockade therapy in GBM (Tomaszewski et al., 2022). Likewise, MAPK–ERK and PI3K–Akt–mTOR integrate neurotransmitter- and cytokine-derived signals to regulate proliferation, metabolism, migration and survival across neurons, glioma cells and immune cells (Sweatt, 2004; Laplante and Sabatini, 2012; Roskoski, 2012; Manning and Toker, 2017). Among these computational hubs, STAT3 occupies a particularly prominent position in GBM, functioning downstream of multiple inflammatory cytokines while also mediating the IL-11-driven astrocytic suppressive program and coordinating tumor proliferation, stemness and immune suppression (de la Iglesia et al., 2008; Yu et al., 2014; Priego et al., 2018). NF-κB signaling integrates inflammatory and stress signaling across neural and immune systems (Hayden and Ghosh, 2012; Dresselhaus and Meffert, 2019) and plays critical roles in sustaining glioma stem-like cells (GSCs), which fuel tumor recurrence and drug resistance (Cahill et al., 2016).

Certain molecular systems that contribute to the dynamic plasticity (Table 1D) of neural and immunological synapses also play key roles in GBM progression. TNF-α and IL-1β regulate homeostatic synaptic scaling, neuronal excitability and inflammatory signaling while simultaneously controlling immune-cell activation and cytokine production, thereby coupling chronic neuroinflammation to persistent remodeling of the tumor microenvironment (Beattie et al., 2002; Stellwagen et al., 2005; Tarassishin et al., 2014). Recent evidence implicates the role of TNF-α in maintaining GSC self-renewal (Zhang et al., 2024). Likewise, immune checkpoint pathways including PD-1–PD-L1, discussed in Section “4.2 Molecular crosstalk across neural, immune and neuron-glioblastoma synapses,” establish chronically immunosuppressive immune synapses while accumulating evidence indicates additional roles in regulating neuronal excitability and neural synaptic plasticity (Yokosuka et al., 2012; Zhao et al., 2023). Collectively, these adaptive hubs provide glioblastoma with the capacity to continuously remodel both neural and immune networks, thereby sustaining tumor growth, immune evasion and therapeutic resistance.

Of note, molecular hubs operating across neural and immune synapses have also been characterized in other forms of malignant glioma. IDH-mutant gliomas accumulate the oncometabolite D-2-hydroxyglutarate (D-2HG), which acts not as a neurotransmitter but as a diffusible immunometabolic disruptor: D-2HG inhibits α-ketoglutarate-dependent dioxygenases, alters chromatin accessibility, and impairs CD8^+^ T cell activation, NK cell cytotoxicity, and stimulator-of-interferon-genes (STING) pathway signaling at concentrations achieved in the tumor microenvironment (Bunse et al., 2018; Notarangelo et al., 2022). Mutant-IDH1/2 inhibition with vorasidenib normalizes tumor 2-HG and was the first targeted therapy to demonstrate progression-free-survival benefit in IDH-mutant glioma (INDIGO; NCT04164901) (Mellinghoff et al., 2023) providing a rationale for combination with immunotherapy in this molecular subgroup.

Importantly, although these shared molecular hubs mediate neuro-immune crosstalk in health and in the TME, it is important to re-emphasize that bidirectional, structurally-defined neuro-immune synaptic signaling in the GBM microenvironment has not yet been demonstrated experimentally. Moreover, several of the interactions discussed here have been characterized in other tissues, tumor types, or in neuroinflammatory models rather than in glioblastoma – for example, connexin-43 gap junctions at the immunological synapse were defined in dendritic-cell–T-cell systems, whereas semaphorin- and ephrin-dependent neuroimmune signaling has largely been delineated in developmental and inflammatory contexts. Consequently, the synaptic hub framework presented here should be viewed as an operational model rather than a definitive molecular atlas. It deliberately integrates established physiology with emerging glioblastoma biology to identify candidate points of convergence between neural and immune signaling. As with any conceptual framework, it is necessarily incomplete because the underlying neuroimmune circuitry of GBM remains incompletely resolved. We therefore anticipate that additional hubs, signaling pathways and structurally defined neuroimmune synapses will emerge as higher-resolution spatial, functional and connectomic studies further define the architecture of the glioblastoma neuroimmune network.

Finally, while several other cell-cell interactions within the GBM microenvironment exhibit synapse-like features (section “4.1 Scales for comparison of neural and immune synapses”), many await direct experimental demonstration. Whether tumor cells of different cellular states form synaptic contacts with each other, and whether neurons form direct synaptic contacts with tumor-associated macrophages (TAMs), are notable open questions. Although gap-junction-mediated electrical and metabolic coupling between GBM cells is well established, bona fide chemical synapses between distinct malignant cellular states–for example, NEU/NPC-like and MES/AC-like cells–have not yet been demonstrated. These states are defined primarily by transcriptional profiles: NEU/NPC-like tumor cells are enriched for neuronal and synaptic programs, preferentially receive neuronal synaptic input and exhibit neural activity-responsive Ca^2+^ signaling, but do not recapitulate the morphology or intrinsic electrophysiological properties of mature neurons (Neftel et al., 2019; Venkataramani et al., 2019, 2022). Indeed, existing evidence for neuron–glioma chemical synapses overwhelmingly places the malignant cell postsynaptically. Similarly, no bona fide neuron-to-TAM synapse has yet been demonstrated in GBM. Nonetheless, in the healthy brain microglia form structured somatic purinergic junctions that surveil neuronal activity (Cserép et al., 2019), and myeloid cells express receptors for glutamate, GABA, catecholamines and purines (Shanshiashvili et al., 2017; Mohammadpour et al., 2019; Ruiz-Rodríguez et al., 2023). Neuronal activity could therefore influence TAM phenotype through non-synaptic transmitter release even in the absence of direct neuron–TAM synapses; whether more spatially organized contacts occur in GBM remains unknown.

### 4.4 Neural activity as a common mediator of GBM progression and local immunosuppression in GBM

The shared molecular hubs between neural and immune synapses, and the discovery of neuron-GBM synapses, raise the possibility that neural activity may influence both GBM progression and local immunosuppression within the TME through partially overlapping communication pathways. Recent work provides experimental and clinical evidence for this hypothesis. First, activity-dependent factors NLGN3, BDNF, and glutamate, secreted at NGS, are present at concentrations sufficient to modulate T cell and myeloid-cell function *in vitro* (Mancusi and Monje, 2023). Second, spatial profiling shows that synaptically active, NPC-like-rich tumor regions are systematically associated with myeloid populations bearing immunosuppressive transcriptional signatures and with reduced T cell infiltration (Friebel et al., 2020; Klemm et al., 2020; Pombo Antunes et al., 2021; Greenwald et al., 2024). Third, network-level interventions that reduce neuronal activity including AMPA-receptor blockade with perampanel have begun to show effects on both tumor proliferation and tumor immune cell composition in preclinical models (Lange et al., 2019; Venkataramani et al., 2019; Venkatesh et al., 2019). However, we underscore that the link between neuronal activity and local immunosuppression in glioblastoma is, at present, a hypothesis supported by spatial association rather than by causal experiment. Direct demonstration of activity-driven immunosuppression *in vitro* and *in vivo* remains a critical experimental gap.

## 5 Glioblastoma therapeutic strategies targeting neural/immune synaptic hubs

### 5.1 Clinically advanced, hub-centered interventions

Several interventions targeting the neural/immune synaptic hubs detailed previously are already in clinical evaluation or approved for use in glioblastoma, although, in many cases, robust biological validation of synaptic hubs has not consistently translated into clinical efficacy. Perampanel, an FDA- and EMA-approved noncompetitive AMPA-receptor antagonist for refractory epilepsy, suppresses NGS function and reduces tumor proliferation and connectivity in preclinical glioblastoma models, with synergy with temozolomide and radiotherapy and rescue of radiation-induced connectivity gains (Venkataramani et al., 2019; Salmaggi et al., 2021; Tetzlaff et al., 2025). The PerSurge/NOA-30 phase II trial (EU-CT 2023-503938-52-00) provides the first prospective test of presurgical perampanel in recurrent glioblastoma, with biological readouts including tumor connectivity and proliferation (Heuer et al., 2024). Perampanel additionally controls tumor-associated epilepsy– which itself contributes to activity-dependent growth–providing a rare opportunity for combined symptomatic and disease-modifying effect. Dordaviprone (ONC201), a DRD2 antagonist and ClpP agonist, has demonstrated radiographic responses in H3K27M-mutant DMG and received FDA accelerated approval following the ACTION trial program (NCT05580562) (Arrillaga-Romany et al., 2024), with confirmatory survival data pending. Its mechanism connects neurotransmitter signaling (dopaminergic) to mitochondrial-protease activation, illustrating cross-pathway exploitation in cancer neuroscience.

These results must also be read against the limitations that condition benefit in practice. Perampanel, although mechanistically compelling, has no completed efficacy trial for tumor control in glioblastoma; PerSurge/NOA-30 is an early-phase, biomarker-focused window-of-opportunity study designed to test target engagement rather than to establish survival benefit. Dordaviprone received accelerated approval on the basis of radiographic response rate in a pooled analysis of non-randomized studies, with confirmatory survival data from ACTION still pending. Across these programs, benefit is further conditioned by patient-selection factors – MGMT promoter methylation, IDH and H3 status, mismatch-repair deficiency, and extent of resection and prior therapy – and by resistance mechanisms that are themselves network properties, including antigen and target loss under therapeutic pressure, transcriptional-state plasticity, and repair of the tumor-microtube network from less-connected reservoir cells after focal disruption. The consistent failure of these agents in unselected populations argues that biomarker-enriched trial designs are a prerequisite for detecting activity in this disease rather than a refinement to be added later.

A further and underappreciated limitation of neurotransmitter-directed therapy is that its targets are physiological signaling systems, so target engagement carries an intrinsic liability for normal neuronal function. AMPA-receptor blockade with perampanel is dose-limited by CNS adverse effects including dizziness, somnolence, gait disturbance and neuropsychiatric symptoms, which constrain the exposures achievable in patients relative to those used in preclinical models, and which are compounded in a population already burdened by tumor-associated epilepsy, corticosteroid exposure and cognitive impairment. Muscarinic and dopaminergic antagonism carry their own cognitive, motor and endocrine liabilities, and systemic modulation of adenosinergic or adrenergic signaling has cardiovascular and immunological consequences that may themselves oppose antitumor immunity. Establishing a therapeutic window will therefore depend on tumor-selective delivery, on exploiting the activity-dependence of the tumor-promoting signal, or on targeting downstream effectors that are tumor-restricted, rather than on global pharmacological blockade of neurotransmission. Quantifying this window–in particular, whether doses that measurably reduce tumor connectivity are tolerable long term–is an explicit objective of current window-of-opportunity trial designs.

A Phase III open-label clinical trial of the αvβ3 inhibitor cilengitide (CENTRIC; NCT00689221) found no improvement in median survival over temozolomide chemotherapy alone (Stupp et al., 2014). The randomized trials of PD-1 blockade have been negative in newly diagnosed and recurrent glioblastoma, including CheckMate 143 (recurrent; NCT02017717), CheckMate 498 (unmethylated-MGMT; NCT02617589) and CheckMate 548 (methylated-MGMT; NCT02667587), with benefit confined at best to selected subsets such as mismatch-repair-deficient tumors (Das et al., 2023; Negm et al., 2025). Combination checkpoint blockade (anti-CTLA-4 plus anti-PD-1) has been shown to significantly improve tumor-free survival in immunocompetent murine GBM models (Reardon et al., 2020), suggesting that combination therapy disrupting multiple hubs may overcome the extensive redundancy and plasticity of the neuron-tumor-immune signaling network in GBM.

Tumor-treating fields (TTFs) deliver low-intensity alternating electric fields that disrupt mitotic spindle assembly and have been shown in the EF-14 trial (NCT00916409) to improve overall survival when added to maintenance temozolomide (Stupp et al., 2017). Recent work suggests TTFs also modulate immune cell function (Ghiaseddin et al., 2020; Barsheshet et al., 2022; Chen et al., 2022a), thus potentially acting through multiple, shared synaptic hubs to confer therapeutic benefit in GBM.

It is notable that most clinically advanced strategies target the immune synapse or tumor cells directly rather than the neuron–glioblastoma synapse. Several factors explain this: the NGS field is young (bona fide synapses were demonstrated only from 2019), so dedicated clinical programs are nascent; selectively suppressing NGS without disrupting physiological neurotransmission is pharmacologically difficult, so current efforts largely repurpose approved agents (e.g., perampanel) or seek to exploit activity-dependence; and clinical and manufacturing infrastructure is far more mature for immunotherapy and CAR-T. Consequently, NGS have received extensive preclinical but limited clinical attention – a translational gap that is, in our view, a central opportunity of the cancer-neuroscience framework.

### 5.2 Emerging GBM clinical strategies targeting neural/immune synaptic hubs

CAR T cell therapy in glioblastoma has entered clinical proof-of-concept. GD2-targeted CAR T cells (NCT04196413) produced radiographic responses in pediatric H3K27M-mutant DMG (Majzner et al., 2022; Monje et al., 2025), and bivalent EGFR/IL-13Rα2-targeted CAR T cells (NCT05168423) have shown neurosurgical and radiographic activity in adult glioblastoma (Bagley et al., 2024). CAR T cells engineered to target EGFR variant III tumor-specific antigen, as well as wild-type EGFR protein (CARv3-TEAM-E) resulted in rapid radiographic tumor regression, although the responses were transient in two of three patients (INCIPIENT trial; NCT05660369) (Choi et al., 2024). Persistence and depth of response remain limited, in part because of the immune-synapse-level dysfunction and trogocytosis-mediated antigen loss reviewed above (Hamieh et al., 2019). Strategies to address these limitations including dual-targeting constructs, armored CARs expressing cytokines or checkpoint-blocking domains, logic-gated circuits, and synthetic Notch receptors are in active development.

CD47 blockade with magrolimab, TTI-621, and related agents aims to overcome the SIRPα-mediated phagocytic checkpoint and is in evaluation in CNS tumors (Gholamin et al., 2017; Hutter et al., 2019). Anti-IL-11 antibodies and STAT3 inhibitors are early-stage candidates motivated directly by the IL-11–STAT3–TRAIL axis (Faust Akl et al., 2025). Bispecific T cell engagers (BiTEs) targeting EGFRvIII and other antigens provide a synthetic immune synapse and are in clinical evaluation.

Focused ultrasound, applied with microbubble contrast, transiently and reversibly opens the blood–brain barrier and represents a major emerging adjunct for delivery of antibodies, CAR T cells, and small molecules into glioblastoma, addressing one of the most persistent translational obstacles in CNS oncology (Lipsman et al., 2018; Mainprize et al., 2019).

Three GBM-specific barriers constrain these emerging strategies. First, antigen heterogeneity: target antigens such as EGFRvIII are expressed heterogeneously and are lost under immune pressure, driving antigen-negative escape – the rationale for dual-/multi-targeting and antigen-spreading approaches. Second, immune exclusion: glioblastoma is characteristically T-cell–poor, with sequestration of naïve T cells in the bone marrow and exclusion of effectors from the tumor (Chongsathidkiet et al., 2018), limiting the substrate on which cellular therapies act. Third, poor persistence: adoptively transferred and endogenous T cells rapidly acquire an exhausted, hypofunctional phenotype in the immunosuppressive microenvironment, curtailing durable responses. Armored CARs, checkpoint-resistant designs, and combination with microenvironment-modifying agents are being developed to counter these specific liabilities.

Nanotechnology-based platforms are also being developed to improve intratumoral delivery and to reprogram the immunosuppressive microenvironment in glioblastoma (Frumento and Ţălu, 2026).

### 5.3 Potential novel synaptic hub-centered strategies for GBM treatment

To avoid conflating aspiration with evidence, we label each strategy below by translational status – preclinically supported/near-term, conceptual/not yet translatable, or modular but early-stage – as indicated in the text.

Several speculative but biologically grounded strategies emerge directly from the network framework. Synapse-disrupting or “synapse-starving” therapies–such as activity-dependent expression cassettes (e.g., c-Fos-driven cytotoxic constructs) *[conceptual – not yet translatable]* –exploit the activity dependence of NGS to selectively target highly active tumor regions; although such constructs have been used preclinically in neuroscience, their translation to oncology remains aspirational. Optogenetic and chemogenetic interventions to silence glioma-innervating neurons are not clinically translatable in current form but provide invaluable mechanistic tools and conceptual proof of principle *[conceptual – not yet translatable]*. Non-invasive neuromodulation strategies *[preclinically supported – near-term]* - transcranial magnetic stimulation, transcranial direct-current stimulation, and deep-brain stimulation represent more tractable analogues and are increasingly used to map glioma functional connectivity (Drexler et al., 2024). Whether these modalities can be employed therapeutically to suppress tumor-promoting activity is an open question but a logical near-term test.

Engineered oncolytic viruses with synaptic-activity-dependent or state-restricted replication, extracellular-vesicle-based payload delivery, and mRNA-based reprogramming approaches *[modular – early-stage]* offer modular platforms for combinatorial multi-target intervention. Ultimately, the network framework predicts that durable disease control will require interventions that simultaneously disrupt activity-dependent tumor-promoting signaling and restore antitumor immune function–a principle most cleanly tested by combinations of perampanel, ONC201, or vorasidenib with checkpoint blockade, CAR T cells, or anti-CD47/anti-IL-11 agents in immunocompetent models.

## 6 Discussion

In this review, we analyze the evidence for disordered neural and immune synaptic communication in glioblastoma to argue that its reconceptualization as a disorder of the neuroimmune connectome provides crucial insight into its pathogenesis and new avenues for therapy. The neural/immune synaptic network model we describe is by no means complete. The activity-dependence of glioma growth through glutamatergic (and, in DMG, GABAergic) synapses is well supported experimentally, and the dysfunction of the immune synapse in GBM is well documented; but the proposition that unites them – that neuronal activity and synaptic communication constitute a single coupled system governing both tumor growth and immune evasion–is currently supported more by shared molecular vocabulary and spatial association than by direct causal demonstration. We therefore present the model as a framework that unifies disparate observations and, importantly, generates falsifiable predictions, while remaining explicit that its central integrative claim awaits direct experimental testing.

How does this neuro-immune synaptic framework integrate with other existing models of GBM biology? Like all cancers, GBM also exhibits key cancer hallmarks including uncontrolled cell proliferation, invasion, angiogenesis and host immune evasion (Hanahan and Weinberg, 2011), and neuronal activity has emerged as a critical regulator of several of these hallmarks (Hanahan and Monje, 2023). As summarized in this review, studies across cellular and murine glioma models over the last decade have identified neuron-glioma synaptic interactions as a key mechanism by which neuronal activity influences tumor progression. In parallel, single-cell and spatial transcriptomic studies have identified dynamic malignant cellular states and multicellular niches in which tumor, neural, vascular and immune populations coexist, and also profiled the immunosuppressive TME. Finally, an increasing body of evidence supports the existence of shared molecular vocabulary and synaptic design principles across the nervous and immune systems. Viewed across these dimensions, synaptic hubs represent molecular nodes within an interaction network superimposed upon GBM cellular states and spatial niches, potentially coupling neuronal activity and immune state to multiple canonical cancer hallmarks. This additional layer of complexity currently raises more questions than it answers. Emerging evidence indicates that certain cellular states dominate the tumor’s infiltrative margin and receive abundant synaptic input while others coordinate intra-tumor communication (Venkataramani et al., 2022), and that combined targeting of multiple GSC cellular states disrupts malignant progression (Lu et al., 2025). However, we still lack an integrated understanding of how neuro/immune synaptic signaling influences or is influenced by tumor cellular state and spatial organization. Do distinct GBM cellular states differ in their dependence on neural or immune synaptic input? Do particular spatial niches generate distinct synaptic architectures? Can disrupting a shared synaptic hub simultaneously shift malignant cell state, restore antitumor immunity and suppress multiple cancer hallmarks, or will network redundancy simply redirect signaling through alternative hubs? Answering these questions will require combining functional perturbation of neuroimmune signaling with single-cell and spatial profiling, rather than studying each layer independently.

Importantly, several neural activity-dependent and immune interactions operate through non-synaptic mechanisms (such as through paracrine factors or the extracellular matrix). The tumor itself is composed of multiple other cellular types whose intercommunication is in part mediated by structured interfaces that may share some, but not all, features of classical neural/immune synapses. Also, identifying shared molecular hubs across neural and immune synapses in complex tumor networks provides limited information on causal ordering–specifically, which interactions contribute to tumor initiation and which emerge during progression or orchestrate relapse. A shared hub on the molecular scale does not establish that it couples networks at the cellular or circuit scale; shared hubs may operate independently in different cell types and on different timescales. Despite these limitations, we posit that this model nevertheless offers a useful compromise between complexity and tractability, for it enables understanding of how activity-dependent signals are transmitted across tumor, neural and immune compartments through structured interfaces amenable to experimental testing and therapeutic targeting. The framework therefore does not require every interaction within the GBM ecosystem to satisfy a strict structural definition of a synapse; rather, it asks whether principles derived from synaptic organization provide a useful level of abstraction for understanding how information is transmitted and integrated across the tumor ecosystem.

Glioma models most commonly used to study neuron-glioma interactions differ fundamentally from those used to characterize glioma immune microenvironments, presenting a major challenge to experimentally testing and integrating evidence for neuro-immune synaptic crosstalk. Studies characterizing neuron-glioma synapses and activity-dependent tumor growth have been undertaken predominantly in rodent xenograft models (human glioma cells transplanted orthotopically into immunodeficient mice), which preserve neural circuitry but eliminate adaptive immunity. GBM immunobiology, on the other hand, has been studied largely in syngeneic models such as SB28, GL261, and CT-2A, which preserve immunity but use cancer lines whose representativeness for human glioblastoma is variable, and which are typically implanted into mice with limited consideration of circuit architecture (Letchuman et al., 2022; Yadav and Purow, 2024). Genetically engineered mouse models induced by viral or in utero electroporation of oncogenic constructs preserve both circuitry and immunity but have their own limitations of background and tractability. Humanized-immune-system patient-derived xenografts and brain-organoid co-cultures with iPSC-derived microglia and T cells are emerging as complementary platforms for human-relevant neural–immune–tumor coupling (Linkous and Fine, 2020).

A further experimental challenge, particularly *in vivo*, is the requirement for platforms that allow simultaneous interrogation of neuronal activity, tumor cell behavior, and immune cell signaling with single-cell and, ideally, synaptic-scale resolution. Approaches that could be potentially multiplexed for this purpose in immunocompetent glioma models include genetically encoded fluorescent sensor readouts of cellular signaling [ex: Ca^2+^ imaging (Chen et al., 2013), GRAB sensors (Sun et al., 2020)], *in vivo* two-photon and super-resolution microscopy (Sigal et al., 2015), serially sampling the transcriptome of live cells [Liveseq (Chen et al., 2022b)], genetically barcoding specific neuron and immune cell subsets for single-cell multiomics with high spatiotemporal resolution [RABIDSeq (Clark et al., 2021)], and perturbation toolkits [optogenetics (Boyden et al., 2005; Månsson et al., 2026) and chemogenetics (Armbruster et al., 2007; Guo et al., 2024)].

The framing of GBM as a disorder of coupled immune and synaptic networks, and of neuronal activity as a common regulator of both, has important therapeutic implications. Targeting either neuronal or immune signaling may have unintended consequences for the other, which may not always be beneficial; while targeting peritumoral neuronal activity or downstream mechanisms may restore conditions permissive for immune activity, while immunotherapy applied without addressing tumor-promoting neuronal input may be undermined by ongoing activity-dependent immunosuppression. Simultaneous disruption of activity-dependent tumor-promoting signaling and restoration of antitumor immunity could produce effects greater than the sum of the parts.

In conclusion, the neuro-immune synaptic network model offers a coherent, though incomplete, conceptual framework that unifies otherwise separate observations in glioblastoma and yields testable therapeutic predictions. Its central and still-unproven prediction–that durable disease control will require simultaneously constraining activity-dependent tumor-promoting signaling and restoring antitumor immunity (a “double-hit” strategy) – should be regarded as a hypothesis to be validated in immunocompetent models and early-phase trials rather than as an established therapeutic principle. Demonstrating concurrent modulation of neural activity and antitumor immunity, with benefit exceeding either alone, is the pivotal experiment the framework demands.

## References

[B1] Aabedi A. A. Young J. S. Zhang Y. Ammanuel S. Morshed R. A. Dalle Ore C. et al. (2022). Association of neurological impairment on the relative benefit of maximal extent of resection in chemoradiation-treated newly diagnosed isocitrate dehydrogenase wild-type glioblastoma. *Neurosurgery* 90, 124–130. 10.1227/neu.0000000000001753 34982879 PMC9514750

[B2] Adams J. P. Dudek S. M. (2005). Late-phase long-term potentiation: Getting to the nucleus. *Nat. Rev. Neurosci.* 6, 737–743. 10.1038/nrn1749 16136174

[B3] Allard D. Allard B. Stagg J. (2020). On the mechanism of anti-CD39 immune checkpoint therapy. *J. Immunother Cancer* 8:e000186. 10.1136/jitc-2019-000186 32098829 PMC7057429

[B4] Allgood J. E. Johnson T. Pullan J. E. (2026). SPARCL1 enrichment at the glioblastoma invasive front is consistent with synaptogenic and angiogenic tumor niches. *Intern. J. Mol. Sci.* 27:4017. 10.3390/ijms27094017 42123596 PMC13164218

[B5] Armbruster B. N. Li X. Pausch M. H. Herlitze S. Roth B. L. (2007). Evolving the lock to fit the key to create a family of G protein-coupled receptors potently activated by an inert ligand. *Proc. Natl. Acad. Sci. U. S. A.* 104, 5163–5168. 10.1073/pnas.0700293104 17360345 PMC1829280

[B6] Arrillaga-Romany I. Gardner S. L. Odia Y. Aguilera D. Allen J. E. Batchelor T. (2024). ONC201 (dordaviprone) in recurrent H3 K27M-mutant diffuse midline glioma. *J. Clin. Oncol.* 42, 1542–1552. 10.1200/JCO.23.01134 38335473 PMC11095894

[B7] Asano Y. Ma C. Y. Limback-Stokin M. M. Rochussen A. M. Stinchcombe J. C. Griffiths G. M. (2025). Nuclear polarization to the immune synapse facilitates an early transcriptional burst. *Sci. Immunol.* 10:eadt5909. 10.1126/sciimmunol.adt5909 40971450

[B8] Bagley S. J. Logun M. Fraietta J. A. Wang X. Desai A. S. Bagley L. J. (2024). Intrathecal bivalent CAR T cells targeting EGFR and IL13Rα2 in recurrent glioblastoma: Phase 1 trial interim results. *Nat. Med.* 30, 1320–1329. 10.1038/s41591-024-02893-z 38480922 PMC13123313

[B9] Barron T. Yalçın B. Su M. Byun Y. G. Gavish A. Geraghty A. C. (2025). GABAergic neuron-to-glioma synapses in diffuse midline gliomas. *Nature* 639, 1060–1068. 10.1038/s41586-024-08579-3 39972132 PMC11946904

[B10] Barsheshet Y. Brant B. Voloshin T. Volodin A. Koren L. Koltun B. et al. (2022). Abstract 1305: Tumor Treating Fields (TTFields) promote a pro-inflammatory phenotype in macrophages. *Cancer Res.* 82, 1305–1305. 10.1158/1538-7445.Am2022-1305

[B11] Beattie E. C. Stellwagen D. Morishita W. Bresnahan J. C. Ha B. K. Von Zastrow M. et al. (2002). Control of synaptic strength by glial TNFalpha. *Science* 295, 2282–2285. 10.1126/science.1067859 11910117

[B12] Ben-Ari Y. (2002). Excitatory actions of GABA during development: The nature of the nurture. *Nat. Rev. Neurosci.* 3, 728–739. 10.1038/nrn920 12209121

[B13] Berghoff A. S. Kiesel B. Widhalm G. Wilhelm D. Rajky O. Kurscheid S. (2017). Correlation of immune phenotype with IDH mutation in diffuse glioma. *Neuro Oncol.* 19, 1460–1468. 10.1093/neuonc/nox054 28531337 PMC5737620

[B14] Bernstock J. D. Mehari M. Gerstl J. V. E. Meredith D. M. Valdes P. A. Heesen P. et al. (2025). Gabapentinoids confer survival benefit in human glioblastoma. *Nat. Commun.* 16:4483. 10.1038/s41467-025-59614-4 40374596 PMC12081740

[B15] Binder D. K. Nagelhus E. A. Ottersen O. P. (2012). Aquaporin-4 and epilepsy. *Glia* 60, 1203–1214. 10.1002/glia.22317 22378467

[B16] Birdsall H. H. (1991). Induction of ICAM-1 on human neural cells and mechanisms of neutrophil-mediated injury. *Am. J. Pathol.* 139, 1341–1350. PMC1886480 1684266

[B17] Boulanger L. M. (2009). Immune proteins in brain development and synaptic plasticity. *Neuron* 64, 93–109. 10.1016/j.neuron.2009.09.001 19840552

[B18] Boyden E. S. Zhang F. Bamberg E. Nagel G. Deisseroth K. (2005). Millisecond-timescale, genetically targeted optical control of neural activity. *Nat. Neurosci.* 8, 1263–1268. 10.1038/nn1525 16116447

[B19] Buckingham S. C. Campbell S. L. Haas B. R. Montana V. Robel S. Ogunrinu T. (2011). Glutamate release by primary brain tumors induces epileptic activity. *Nat. Med.* 17, 1269–1274. 10.1038/nm.2453 21909104 PMC3192231

[B20] Bunse L. Pusch S. Bunse T. Sahm F. Sanghvi K. Friedrich M. (2018). Suppression of antitumor T cell immunity by the oncometabolite (R)-2-hydroxyglutarate. *Nat. Med.* 24, 1192–1203. 10.1038/s41591-018-0095-6 29988124

[B21] Cahill K. E. Morshed R. A. Yamini B. (2016). Nuclear factor-κB in glioblastoma: Insights into regulators and targeted therapy. *Neuro Oncol.* 18, 329–339. 10.1093/neuonc/nov265 26534766 PMC4767244

[B22] Campbell S. L. Robel S. Cuddapah V. A. Robert S. Buckingham S. C. Kahle K. T. et al. (2015). GABAergic disinhibition and impaired KCC2 cotransporter activity underlie tumor-associated epilepsy. *Glia* 63, 23–36. 10.1002/glia.22730 25066727 PMC4237714

[B23] Cascio G. Martín-Cófreces N. B. Rodríguez-Frade J. M. López-Cobo S. Lanzavecchia A. (2015). CXCL12 regulates through JAK1 and JAK2 formation of productive immunological synapses. *J. Immunol.* 194, 5509–5519. 10.4049/jimmunol.1402419 25917087

[B24] Chanda S. Hale W. D. Zhang B. Wernig M. Südhof T. C. (2017). Unique versus redundant functions of neuroligin genes in shaping excitatory and inhibitory synapse properties. *J. Neurosci.* 37, 6816–6836. 10.1523/jneurosci.0125-17.2017 28607166 PMC5518416

[B25] Chao M. P. Weissman I. L. Majeti R. (2012). The CD47-SIRPα pathway in cancer immune evasion and potential therapeutic implications. *Curr. Opin. Immunol.* 24, 225–232. 10.1016/j.coi.2012.01.010 22310103 PMC3319521

[B26] Chaudhari A. Padmar J. Awathale S. Goyal S. Nakhate K. Sherikar A. (2025). From glial cells to pain pathways: ICAM-1 as a central player in neuroinflammation and neuropathy. *Discover Neurosci.* 20:5. 10.1186/s13064-025-00201-0

[B27] Chen T.-W. Wardill T. J. Sun Y. Pulver S. R. Renninger S. L. Baohan A. et al. (2013). Ultrasensitive fluorescent proteins for imaging neuronal activity. *Nature* 499, 295–300. 10.1038/nature12354 23868258 PMC3777791

[B28] Chen D. Le S. B. Hutchinson T. E. Calinescu A.-A. Sebastian M. Jin D. et al. (2022a). Tumor treating fields dually activate STING and AIM2 inflammasomes to induce adjuvant immunity in glioblastoma. *J. Clin. Invest.* 132:e149258. 10.1172/JCI149258 35199647 PMC9012294

[B29] Chen W. Guillaume-Gentil O. Rainer P. Y. Gäbelein C. G. Saelens W. Gardeux V. et al. (2022b). Live-seq enables temporal transcriptomic recording of single cells. *Nature* 608, 733–740. 10.1038/s41586-022-05046-9 35978187 PMC9402441

[B30] Choi B. D. Gerstner E. R. Frigault M. J. Leick M. B. Mount C. W. Balaj L. (2024). Intraventricular CARv3-TEAM-E T cells in recurrent glioblastoma. *N. Engl. J. Med.* 390, 1290–1298. 10.1056/NEJMoa2314390 38477966 PMC11162836

[B31] Chongsathidkiet P. Jackson C. Koyama S. Loebel F. Cui X. Farber S. H. (2018). Sequestration of T cells in bone marrow in the setting of glioblastoma and other intracranial tumors. *Nat. Med.* 24, 1459–1468. 10.1038/s41591-018-0135-2 30104766 PMC6129206

[B32] Christopherson K. S. Ullian E. M. Stokes C. C. Mullowney C. E. Hell J. W. Agah A. et al. (2005). Thrombospondins are astrocyte-secreted proteins that promote CNS synaptogenesis. *Cell* 120, 421–433. 10.1016/j.cell.2004.12.020 15707899

[B33] Ciceroni C. Bonelli M. Mastrantoni E. Niccolini C. Laurenza M. Larocca L. M. (2013). Type-3 metabotropic glutamate receptors regulate chemoresistance in glioma stem cells, and their levels are inversely related to survival in patients with malignant gliomas. *Cell Death Differ.* 20, 396–407. 10.1038/cdd.2012.150 23175182 PMC3569992

[B34] Claes A. Idema A. J. Wesseling P. (2007). Diffuse glioma growth: A guerilla war. *Acta Neuropathol.* 114, 443–458. 10.1007/s00401-007-0293-7 17805551 PMC2039798

[B35] Clark I. C. Gutiérrez-Vázquez C. Wheeler M. A. Li Z. Rothhammer V. Linnerbauer M. et al. (2021). Barcoded viral tracing of single-cell interactions in central nervous system inflammation. *Science* 372:eabf1230. 10.1126/science.abf1230 33888612 PMC8157482

[B36] Cserép C. Pósfai B. Lénárt N. Fekete R. László Z. I. Lele Z. (2019). Microglia monitor and protect neuronal function through specialized somatic purinergic junctions. *Science* 367, 528–537. 10.1126/science.aax6752 31831638

[B37] Cuddapah V. A. Robel S. Watkins S. Sontheimer H. (2014). A neurocentric perspective on glioma invasion. *Nat. Rev. Neurosci.* 15, 455–465. 10.1038/nrn3765 24946761 PMC5304245

[B38] Curry R. N. Aiba I. Meyer J. Lozzi B. Ko Y. McDonald M. F. (2023). Glioma epileptiform activity and progression are driven by IGSF3-mediated potassium dysregulation. *Neuron* 111, 682–695.e9. 10.1016/j.neuron.2023.01.013 36787748 PMC9991983

[B39] Das A. Tabori U. Sambira Nahum L. C. Collins N. B. Deyell R. Dvir R. et al. (2023). Efficacy of nivolumab in pediatric cancers with high mutation burden and mismatch repair deficiency. *Clin. Cancer Res.* 29, 4770–4783. 10.1158/1078-0432.Ccr-23-0411 37126021 PMC10690097

[B40] Daubon T. Léon C. Clarke K. Andrique L. Salabert L. Darbo E. et al. (2019). Deciphering the complex role of thrombospondin-1 in glioblastoma development. *Nat. Commun.* 10:1146. 10.1038/s41467-019-08480-y 30850588 PMC6408502

[B41] Day B. W. Stringer B. W. Al-Ejeh F. Ting M. J. Wilson J. Ensbey K. S. et al. (2013). EphA3 maintains tumorigenicity and is a therapeutic target in glioblastoma multiforme. *Cancer Cell* 23, 238–248. 10.1016/j.ccr.2013.01.007 23410976

[B42] De Biase L. M. Nishiyama A. Bergles D. E. (2010). Excitability and synaptic communication within the oligodendrocyte lineage. *J. Neurosci.* 30, 3600–3611. 10.1523/JNEUROSCI.6000-09.2010 20219994 PMC2838193

[B43] de la Iglesia N. Konopka G. Lim K. L. Nutt C. L. Bromberg J. F. Frank D. A. et al. (2008). Deregulation of a STAT3-interleukin 8 signaling pathway promotes human glioblastoma cell proliferation and invasiveness. *J. Neurosci.* 28, 5870–5878. 10.1523/jneurosci.5385-07.2008 18524891 PMC2700037

[B44] Deisseroth K. Mermelstein P. G. Xia H. Tsien R. W. (2003). Signaling from synapse to nucleus: The logic behind the mechanisms. *Curr. Opin. Neurobiol.* 13, 354–365. 10.1016/s0959-4388(03)00076-x 12850221

[B45] Deisseroth K. Singla S. Toda H. Monje M. Palmer T. D. Malenka R. C. (2004). Excitation-neurogenesis coupling in adult neural stem/progenitor cells. *Neuron* 42, 535–552. 10.1016/s0896-6273(04)00266-1 15157417

[B46] do Carmo A. Patricio I. Cruz M. T. Carvalheiro H. Oliveira C. R. Lopes M. C. (2010). CXCL12/CXCR4 promotes motility and proliferation of glioma cells. *Cancer Biol. Ther.* 9, 56–65. 10.4161/cbt.9.1.10342 19923906

[B47] Draganić K. Isaev S. Pototschnig I. Valcanover D. Pfneissl J. Stadler M. et al. (2026). Fibroblast-derived thrombospondin-1 shapes macrophage polarization in advanced human co-culture models. *bioRxiv* [Preprint] 10.64898/2026.05.28.728363

[B48] Dresselhaus E. C. Meffert M. K. (2019). Cellular specificity of NF-κB function in the nervous system. *Front. Immunol.* 10:1043. 10.3389/fimmu.2019.01043 31143184 PMC6520659

[B49] Drexler R. Khatri R. Sauvigny T. Mohme M. Maire C. L. Ryba A. (2024). A prognostic neural epigenetic signature in high-grade glioma. *Nat. Med.* 30, 1622–1635. 10.1038/s41591-024-02969-w 38760585 PMC11186787

[B50] Dustin M. L. (2008). T-cell activation through immunological synapses and kinapses. *Immunol. Rev.* 221, 77–89. 10.1111/j.1600-065X.2008.00589.x 18275476

[B51] Dustin M. L. (2014). The immunological synapse. *Cancer Immunol. Res.* 2, 1023–1033. 10.1158/2326-6066.CIR-14-0161 25367977 PMC4692051

[B52] Dustin M. L. Colman D. R. (2002). Neural and immunological synaptic relations. *Science* 298, 785–789. 10.1126/science.1076386 12399580

[B53] Dustin M. L. Groves J. T. (2012). Receptor signaling clusters in the immune synapse. *Annu. Rev. Biophys.* 41, 543–556. 10.1146/annurev-biophys-042910-155238 22404679 PMC4000727

[B54] Dustin M. L. Springer T. A. (1989). T-cell receptor cross-linking transiently stimulates adhesiveness through LFA-1. *Nature* 341, 619–624. 10.1038/341619a0 2477710

[B55] Eberhart C. G. Bar E. E. (2020). Spatial enrichment of cellular states in glioblastoma. *Acta Neuropathol.* 140, 85–87. 10.1007/s00401-020-02165-3 32449056 PMC7362280

[B56] Ehtesham M. Winston J. A. Kabos P. Thompson R. C. (2006). CXCR4 expression mediates glioma cell invasiveness. *Oncogene* 25, 2801–2806. 10.1038/sj.onc.1209302 16407848

[B57] Eroglu C. Allen N. J. Susman M. W. O’Rourke N. A. Park C. Y. Ozkan E. et al. (2009). Gabapentin receptor alpha2delta-1 is a neuronal thrombospondin receptor responsible for excitatory CNS synaptogenesis. *Cell* 139, 380–392. 10.1016/j.cell.2009.09.025 19818485 PMC2791798

[B58] Faust Akl C. Andersen B. M. Li Z. Giovannoni F. Diebold M. Sanmarco L. M. (2025). Glioblastoma-instructed astrocytes suppress tumour-specific T cell immunity. *Nature* 643, 219–229. 10.1038/s41586-025-08997-x 40399681 PMC12765214

[B59] Filiano A. J. Xu Y. Tustison N. J. Marsh R. L. Baker W. Smirnov I. (2016). Unexpected role of interferon-γ in regulating neuronal connectivity and social behaviour. *Nature* 535, 425–429. 10.1038/nature18626 27409813 PMC4961620

[B60] Franovic A. Elliott K. C. Seguin L. Camargo M. F. Weis S. M. Cheresh D. A. (2015). Glioblastomas require integrin αvβ3/PAK4 signaling to escape senescence. *Cancer Res.* 75, 4466–4473. 10.1158/0008-5472.Can-15-0988 26297735 PMC4631634

[B61] Friebel E. Kapolou K. Unger S. (2020). Single-Cell mapping of human brain cancer reveals tumor-specific instruction of tissue-invading leukocytes. *Cell* 181, 1626–1642.e20. 10.1016/j.cell.2020.04.055 32470397

[B62] Friedl P. Gunzer M. (2001). Interaction of T cells with APCs: The serial encounter model. *Trends Immunol.* 22, 187–191. 10.1016/s1471-4906(01)01869-5 11274922

[B63] Frumento D. Ţălu Ş (2026). Recent developments in nanotechnology-enhanced immunotherapy for glioblastoma. *Immunotherapy* 18, 421–433. 10.1080/1750743x.2026.2674410 42141961 PMC13271326

[B64] Galvao R. P. Kasina A. McNeill R. S. Harbin J. E. Foreman O. Verhaak R. G. W. (2014). Transformation of quiescent adult oligodendrocyte precursor cells into malignant glioma through a multistep reactivation process. *Proc. Natl. Acad. Sci. U. S. A.* 111, E4214–E4223. 10.1073/pnas.1414389111 25246577 PMC4210043

[B65] Gan K. J. Südhof T. C. (2020). SPARCL1 promotes excitatory but not inhibitory synapse formation and function independent of neurexins and neuroligins. *J. Neurosci.* 40, 8088–8102. 10.1523/jneurosci.0454-20.2020 32973045 PMC7574652

[B66] Ganor Y. Levite M. (2014). The neurotransmitter glutamate and human T cells: Glutamate receptors and glutamate-induced direct and potent effects on normal human T cells, cancerous human leukemia and lymphoma T cells, and autoimmune human T cells. *J. Neural Transm.* 121, 983–1006. 10.1007/s00702-014-1167-5 24584970

[B67] Ghiaseddin A. P. Shin D. Melnick K. Tran D. D. (2020). Tumor treating fields in the management of patients with malignant gliomas. *Curr. Treatment Opt. Oncol.* 21:76. 10.1007/s11864-020-00773-5 32734509 PMC7391234

[B68] Gholamin S. Mitra S. S. Feroze A. H. Liu J. Kahn S. A. Zhang M. (2017). Disrupting the CD47-SIRPα anti-phagocytic axis by a humanized anti-CD47 antibody is an efficacious treatment for malignant pediatric brain tumors. *Sci. Transl. Med.* 9:eaaf2968. 10.1126/scitranslmed.aaf2968 28298418

[B69] Gillespie S. M. Kim Y. S. Geraghty A. C. Yalçın B. Mancusi R. Hysinger J. et al. (2025). Neuroligin-3 interaction with CSPG4 regulates normal and malignant glial precursors through PIEZO1. *bioRxiv* [Preprint] 10.1101/2025.07.12.664340 40791371 PMC12338510

[B70] Gillespie S. Kim Y. Geraghty A. Woo P. Monje M. (2021). HGG-05. neuronal activity promotes pediatric high-grade glioma growth through a nlgn3-cspg4 signaling axis. *Neuro-Oncology* 23(Suppl._1), i18–i18. 10.1093/neuonc/noab090.071

[B71] Grakoui A. Bromley S. K. Sumen C. Davis M. M. Shaw A. S. Allen P. M. (1999). The immunological synapse: A molecular machine controlling T cell activation. *Science* 285, 221–227. 10.1126/science.285.5425.221 10398592

[B72] Greenwald A. C. Darnell N. G. Hoefflin R. Simkin D. Mount C. W. Gonzalez Castro L. N. (2024). Integrative spatial analysis reveals a multi-layered organization of glioblastoma. *Cell* 187, 2485–2501.e26. 10.1016/j.cell.2024.03.029 38653236 PMC11088502

[B73] Guo M. Yuan Z. Jin X. Li X. Deng Y. Zhou S. et al. (2025). Inhibition of ICAM1 diminishes stemness and enhances antitumor immunity in glioblastoma via β-catenin/PD-L1 signaling. *Nat. Commun.* 16:8642. 10.1038/s41467-025-63796-2 41028719 PMC12484827

[B74] Guo X. Qiu W. Li B. Qi Y. Wang S. Zhao R. et al. (2024). Hypoxia-Induced neuronal activity in glioma patients polarizes microglia by potentiating RNA m6A demethylation. *Clin. Cancer Res.* 30, 1160–1174. 10.1158/1078-0432.Ccr-23-0430 37855702

[B75] Hamieh M. Dobrin A. Cabriolu A. van der Stegen S. J. C. Giavridis T. Mansilla-Soto J. (2019). CAR T cell trogocytosis and cooperative killing regulate tumour antigen escape. *Nature* 568, 112–116. 10.1038/s41586-019-1054-1 30918399 PMC6707377

[B76] Hanahan D. Monje M. (2023). Cancer hallmarks intersect with neuroscience in the tumor microenvironment. *Cancer Cell* 41, 573–580. 10.1016/j.ccell.2023.02.012 36917953 PMC10202656

[B77] Hanahan D. Weinberg R. A. (2011). Hallmarks of cancer: The next generation. *Cell* 144, 646–674. 10.1016/j.cell.2011.02.013 21376230

[B78] Hara T. Chanoch-Myers R. Mathewson N. D. Myskiw C. Atta L. Bussema L. (2021). Interactions between cancer cells and immune cells drive transitions to mesenchymal-like states in glioblastoma. *Cancer Cell* 39, 779–792.e11. 10.1016/j.ccell.2021.05.002 34087162 PMC8366750

[B79] Hausmann D. Hoffmann D. C. Venkataramani V. Jung E. Horschitz S. Tetzlaff S. K. (2022). Autonomous rhythmic activity in glioma networks drives brain tumour growth. *Nature* 613, 179–186. 10.1038/s41586-022-05520-4 36517594

[B80] Hayden M. S. Ghosh S. (2012). NF-κB, the first quarter-century: Remarkable progress and outstanding questions. *Genes Dev.* 26, 203–234. 10.1101/gad.183434.111 22302935 PMC3278889

[B81] Heuer S. Venkataramani V. Kessler T. Hai L. Winkler F. Wick W. (2024). The PerSurge (NOA-30) trial: Perampanel before surgery in recurrent glioblastoma. *BMC Cancer* 24:135. 10.1186/s12885-024-11846-1 38279087 PMC10811925

[B82] Hills K. E. Kostarelos K. Wykes R. C. (2022). Converging mechanisms of epileptogenesis and their insight in glioblastoma. *Front. Mol. Neurosci.* 15:903115. 10.3389/fnmol.2022.903115 35832394 PMC9271928

[B83] Himes B. T. Geiger P. A. Ayasoufi K. Bhargav A. G. Brown D. A. Parney I. F. (2021). Immunosuppression in glioblastoma: Current understanding and therapeutic implications. *Front. Oncol.* 11:770561. 10.3389/fonc.2021.770561 34778089 PMC8581618

[B84] Hogan P. G. Chen L. Nardone J. Rao A. (2003). Transcriptional regulation by calcium, calcineurin, and NFAT. *Genes Dev.* 17, 2205–2232. 10.1101/gad.1102703 12975316

[B85] Hsieh A. L. Ganesh S. Kula T. Irshad M. Ferenczi E. A. Wang W. et al. (2024). Widespread neuroanatomical integration and distinct electrophysiological properties of glioma-innervating neurons. *Proc. Natl. Acad. Sci. U. S. A.* 121:e2417420121. 10.1073/pnas.2417420121 39630872 PMC11648874

[B86] Hu X. Deng Q. Ma L. Li Q. Chen Y. Liao Y. (2020). Meningeal lymphatic vessels regulate brain tumor drainage and immunity. *Cell Res.* 30, 229–243. 10.1038/s41422-020-0287-8 32094452 PMC7054407

[B87] Huang Q. Chen L. Liang J. Huang Q. Sun H. (2022). Neurotransmitters: Potential targets in glioblastoma. *Cancers* 14:3970. 10.3390/cancers14163970 36010960 PMC9406056

[B88] Huang-Hobbs E. Cheng Y. T. Ko Y. Luna-Figueroa E. Lozzi B. Taylor K. R. et al. (2023). Remote neuronal activity drives glioma progression through SEMA4F. *Nature* 619, 844–850. 10.1038/s41586-023-06267-2 37380778 PMC10840127

[B89] Hui E. Cheung J. Zhu J. Su X. Taylor M. J. Wallweber H. A. et al. (2017). T cell costimulatory receptor CD28 is a primary target for PD-1-mediated inhibition. *Science* 355, 1428–1433. 10.1126/science.aaf1292 28280247 PMC6286077

[B90] Hutter G. Theruvath J. Graef C. M. Zhang M. Schoen M. K. Manz E. M. (2019). Microglia are effector cells of CD47-SIRPα antiphagocytic axis disruption against glioblastoma. *Proc. Natl. Acad. Sci. U. S. A.* 116, 997–1006. 10.1073/pnas.1721434116 30602457 PMC6338872

[B91] Jackson C. M. Choi J. Lim M. (2019). Mechanisms of immunotherapy resistance: Lessons from glioblastoma. *Nat. Immunol.* 20, 1100–1109. 10.1038/s41590-019-0433-y 31358997

[B92] Joseph J. V. Magaut C. R. Storevik S. Geraldo L. H. Mathivet T. Latif M. A. et al. (2022). TGF-β promotes microtube formation in glioblastoma through thrombospondin 1. *Neuro Oncol.* 24, 541–553. 10.1093/neuonc/noab212 34543427 PMC8972291

[B93] Jung E. Osswald M. Blaes J. Wiestler B. Sahm F. Schmenger T. (2017). Tweety-homolog 1 drives brain colonization of gliomas. *J. Neurosci.* 37, 6837–6850. 10.1523/JNEUROSCI.3532-16.2017 28607172 PMC6705725

[B94] Kaur S. Roberts D. D. (2024). Emerging functions of thrombospondin-1 in immunity. *Semin. Cell Dev. Biol.* 155(Pt B), 22–31. 10.1016/j.semcdb.2023.05.008 37258315 PMC10684827

[B95] Kioussis D. Pachnis V. (2009). Immune and nervous systems: More than just a superficial similarity? *Immunity* 31, 705–710. 10.1016/j.immuni.2009.09.009 19836266

[B96] Klemm F. Maas R. R. Bowman R. L. (2020). Interrogation of the microenvironmental landscape in brain tumors reveals disease-specific alterations of immune cells. *Cell* 181, 1643–1660.e17. 10.1016/j.cell.2020.05.007 32470396 PMC8558904

[B97] Köhling R. Senner V. Paulus W. Speckmann E. J. (2006). Epileptiform activity preferentially arises outside tumor invasion zone in glioma xenotransplants. *Neurobiol. Dis.* 22, 64–75. 10.1016/j.nbd.2005.10.001 16309916

[B98] Kotecha R. Odia Y. Khosla A. A. Ahluwalia M. S. (2023). Key clinical principles in the management of glioblastoma. *JCO Oncol. Pract.* 19, 180–189. 10.1200/op.22.00476 36638331

[B99] Krishna S. Choudhury A. Keough M. B. Seo K. Ni L. Kakaizada S. et al. (2023). Glioblastoma remodelling of human neural circuits decreases survival. *Nature* 617, 599–607. 10.1038/s41586-023-06036-1 37138086 PMC10191851

[B100] Krummel M. F. Cahalan M. D. (2010). The immunological synapse: A dynamic platform for local signaling. *J. Clin. Immunol.* 30, 364–372. 10.1007/s10875-010-9393-6 20390326 PMC2874029

[B101] Labrakakis C. Patt S. Hartmann J. Kettenmann H. (1998). Glutamate receptor activation can trigger electrical activity in human glioma cells. *Eur. J. Neurosci.* 10, 2153–2162. 10.1046/j.1460-9568.1998.00226.x 9753101

[B102] Lange F. Wesslau K. Porath K. Hornschemeyer M. F. Bergner C. Krause B. J. et al. (2019). AMPA receptor antagonist perampanel affects glioblastoma cell growth and glutamate release in vitro. *PLoS One* 14:e0211644. 10.1371/journal.pone.0211644 30716120 PMC6361447

[B103] Laplante M. Sabatini D. M. (2012). mTOR signaling in growth control and disease. *Cell* 149, 274–293. 10.1016/j.cell.2012.03.017 22500797 PMC3331679

[B104] Lee J. H. Lee J. E. Kahng J. Y. Kim S. H. Park J. S. Yoon S. J. et al. (2018). Human glioblastoma arises from subventricular zone cells with low-level driver mutations. *Nature* 560, 243–247. 10.1038/s41586-018-0389-3 30069053

[B105] Letchuman V. Ampie L. Shah A. H. Brown D. A. Heiss J. D. Chittiboina P. (2022). Syngeneic murine glioblastoma models: Reactionary immune changes and immunotherapy intervention outcomes. *Neurosurg. Focus* 52, E5. 10.3171/2021.11.Focus21556 35104794 PMC10851929

[B106] Li J. Zhu S. Kozono D. Ng K. Futalan D. Shen Y. (2014). Genome-wide shRNA screen revealed integrated mitogenic signaling between dopamine receptor D2 (DRD2) and epidermal growth factor receptor (EGFR) in glioblastoma. *Oncotarget* 5, 882–893. 10.18632/oncotarget.1801 24658464 PMC4011590

[B107] Li M. Ransohoff R. M. (2008). Multiple roles of chemokine CXCL12 in the central nervous system: A migration from immunology to neurobiology. *Prog. Neurobiol.* 84, 116–131. 10.1016/j.pneurobio.2007.11.003 18177992 PMC2324067

[B108] Li Y. Wang Y. Han X. Xu J. Liu E. Cheng J. et al. (2025). Glioma-derived SPARCL1 promotes the formation of peritumoral neuron-glioma synapses. *J. Neurooncol.* 173, 515–526. 10.1007/s11060-025-05007-y 40227556 PMC12170744

[B109] Lindberg N. Jiang Y. Xie Y. Bolouri H. Kastemar M. Olofsson T. (2014). Oncogenic signaling is dominant to cell of origin and dictates astrocytic or oligodendroglial tumor development from oligodendrocyte precursor cells. *J. Neurosci.* 34, 14644–14651. 10.1523/JNEUROSCI.2977-14.2014 25355217 PMC6608431

[B110] Linkous A. Fine H. A. (2020). Generating patient-derived gliomas within cerebral organoids. *STAR Protoc.* 1:100008. 10.1016/j.xpro.2019.100008 33103125 PMC7580125

[B111] Lipsman N. Meng Y. Bethune A. J. Huang Y. Lam B. Masellis M. (2018). Blood-brain barrier opening in Alzheimer’s disease using MR-guided focused ultrasound. *Nat. Commun.* 9:2336. 10.1038/s41467-018-04529-6 30046032 PMC6060168

[B112] Lisman J. Yasuda R. Raghavachari S. (2012). Mechanisms of CaMKII action in long-term potentiation. *Nat. Rev. Neurosci.* 13, 169–182. 10.1038/nrn3192 22334212 PMC4050655

[B113] Liu C. Luo D. Reynolds B. A. Meher G. Katritzky A. R. Lu B. et al. (2011a). Chemokine receptor CXCR3 promotes growth of glioma. *Carcinogenesis* 32, 129–137. 10.1093/carcin/bgq224 21051441 PMC3026840

[B114] Liu C. Sage J. C. Miller M. R. Verhaak R. G. W. Hippenmeyer S. Vogel H. (2011b). Mosaic analysis with double markers reveals tumor cell of origin in glioma. *Cell* 146, 209–221. 10.1016/j.cell.2011.06.014 21737130 PMC3143261

[B115] Louis D. N. Perry A. Wesseling P. Brat D. J. Cree I. A. Figarella-Branger D. et al. (2021). The 2021 WHO classification of tumors of the central nervous system: A summary. *Neuro Oncol.* 23, 1231–1251. 10.1093/neuonc/noab106 34185076 PMC8328013

[B116] Louveau A. Smirnov I. Keyes T. J. Eccles J. D. Rouhani S. J. Peske J. D. (2015). Structural and functional features of central nervous system lymphatic vessels. *Nature* 523, 337–341. 10.1038/nature14432 26030524 PMC4506234

[B117] Lu C. Kang T. Zhang J. Yang K. Liu Y. Song K. et al. (2025). Combined targeting of glioblastoma stem cells of different cellular states disrupts malignant progression. *Nat. Commun.* 16:2974. 10.1038/s41467-025-58366-5 40140646 PMC11947120

[B118] Lutton E. M. Razmpour R. Andrews A. M. Cannella L. A. Son Y.-J. Shuvaev V. V. et al. (2017). Acute administration of catalase targeted to ICAM-1 attenuates neuropathology in experimental traumatic brain injury. *Sci. Rep.* 7:3846. 10.1038/s41598-017-03309-4 28630485 PMC5476649

[B119] MacKenzie G. O’Toole K. K. Moss S. J. Maguire J. (2016). Compromised GABAergic inhibition contributes to tumor-associated epilepsy. *Epilepsy Res.* 126, 185–196. 10.1016/j.eplepsyres.2016.07.010 27513374 PMC5308901

[B120] Madadi A. Wolfart J. Lange F. Brehme H. Linnebacher M. Bräuer A. U. et al. (2021). Correlation between Kir4.1 expression and barium-sensitive currents in rat and human glioma cell lines. *Neurosci. Lett.* 741:135481. 10.1016/j.neulet.2020.135481 33161102

[B121] Madkhali O. A. Moni S. S. Almoshari Y. Sabei F. Y. Safhi A. Y. (2025). Dual role of CXCL10 in cancer progression: Implications for immunotherapy and targeted treatmentı. *Cancer Biol. Ther.* 26:2538962. 10.1080/15384047.2025.2538962 40760734 PMC12326575

[B122] Magnon C. Hall S. J. Lin J. Xue X. Gerber L. Freedland S. J. (2013). Autonomic nerve development contributes to prostate cancer progression. *Science* 341:1236361. 10.1126/science.1236361 23846904

[B123] Mainprize T. Lipsman N. Huang Y. Meng Y. Bethune A. Ironside S. (2019). Blood-Brain barrier opening in primary brain tumors with non-invasive MR-Guided focused ultrasound: A clinical safety and feasibility study. *Sci. Rep.* 9:321. 10.1038/s41598-018-36340-0 30674905 PMC6344541

[B124] Majzner R. G. Ramakrishna S. Yeom K. W. Patel S. Chinnasamy H. Schultz L. M. et al. (2022). GD2-CAR T cell therapy for H3K27M-mutated diffuse midline gliomas. *Nature* 603, 934–941. 10.1038/s41586-022-04489-4 35130560 PMC8967714

[B125] Makranz C. Huguely C. Zhang M. Davis D. Anderson B. Haynes T. et al. (2026). Inhibition of chemokine CXCL12 modulates the tumor microenvironment in glioblastoma and potentiates immune checkpoint inhibitor efficacy in a tissue-specific manner. *Neurooncol. Adv.* 8:vdag118. 10.1093/noajnl/vdag118 42211393 PMC13215089

[B126] Mancusi R. Monje M. (2023). The neuroscience of cancer. *Nature* 618, 467–479. 10.1038/s41586-023-05968-y 37316719 PMC11146751

[B127] Manning B. D. Toker A. (2017). AKT/PKB signaling: Navigating the network. *Cell* 169, 381–405. 10.1016/j.cell.2017.04.001 28431241 PMC5546324

[B128] Månsson L. K. Dickson E. Hao L. Pitenis A. A. Wilson M. Z. (2026). Optogenetic control of the integrated stress response limits glioblastoma invasion. *Cell Biochem. Funct.* 44:e70212. 10.1002/cbf.70212 41987482

[B129] Martin-Manso G. Galli S. Ridnour L. A. Tsokos M. Wink D. A. Roberts D. D. (2008). Thrombospondin 1 promotes tumor macrophage recruitment and enhances tumor cell cytotoxicity of differentiated U937 cells. *Cancer Res.* 68, 7090–7099. 10.1158/0008-5472.Can-08-0643 18757424 PMC2562557

[B130] Mathewson N. D. Ashenberg O. Tirosh I. (2021). Inhibitory CD161 receptor identified in glioma-infiltrating T cells by single-cell analysis. *Cell* 184, 1281–1298.e26. 10.1016/j.cell.2021.01.022 33592174 PMC7935772

[B131] Mellinghoff I. K. van den Bent M. J. Blumenthal D. T. Touat M. Peters K. B. Clarke J. (2023). Vorasidenib in IDH1- or IDH2-Mutant low-grade glioma. *N. Engl. J. Med.* 389, 589–601. 10.1056/NEJMoa2304194 37272516 PMC11445763

[B132] Mendoza-Naranjo A. Bouma G. Pereda C. Ramírez M. Webb K. F. Tittarelli A. et al. (2011). Functional gap junctions accumulate at the immunological synapse and contribute to T cell activation. *J. Immunol.* 187, 3121–3132. 10.4049/jimmunol.1100378 21844382 PMC3173876

[B133] Mercurio L. Ajmone-Cat M. A. Cecchetti S. Ricci A. Bozzuto G. Molinari A. et al. (2016). Targeting CXCR4 by a selective peptide antagonist modulates tumor microenvironment and microglia reactivity in a human glioblastoma model. *J. Exp. Clin. Cancer Res.* 35:55. 10.1186/s13046-016-0326-y 27015814 PMC4807593

[B134] Miller M. J. Safrina O. Parker I. Cahalan M. D. (2004). Imaging the single cell dynamics of CD4+ T cell activation by dendritic cells in lymph nodes. *J. Exp. Med.* 200, 847–856. 10.1084/jem.20041236 15466619 PMC2213293

[B135] Mohammadpour H. MacDonald C. R. Qiao G. Chen M. Dong B. Hylander B. L. et al. (2019). β2 adrenergic receptor-mediated signaling regulates the immunosuppressive potential of myeloid-derived suppressor cells. *J. Clin. Invest.* 129, 5537–5552. 10.1172/JCI129502 31566578 PMC6877316

[B136] Monje M. Borniger J. C. D’Silva N. J. Deneen B. Dirks P. B. Fattahi F. (2020). Roadmap for the emerging field of cancer neuroscience. *Cell* 181, 219–222. 10.1016/j.cell.2020.03.034 32302564 PMC7286095

[B137] Monje M. Mahdi J. Majzner R. Yeom K. W. Schultz L. M. Richards R. M. et al. (2025). Intravenous and intracranial GD2-CAR T cells for H3K27M+ diffuse midline gliomas. *Nature* 637, 708–715. 10.1038/s41586-024-08171-9 39537919 PMC11735388

[B138] Müller S. Kohanbash G. Liu S. J. Alvarado B. Carrera D. Bhaduri A. (2017). Single-cell profiling of human gliomas reveals macrophage ontogeny as a basis for regional differences in macrophage activation in the tumor microenvironment. *Genome Biol.* 18:234. 10.1186/s13059-017-1362-4 29262845 PMC5738907

[B139] Neftel C. Laffy J. Filbin M. G. Hara T. Shore M. E. Rahme G. J. et al. (2019). An integrative model of cellular states, plasticity, and genetics for glioblastoma. *Cell* 178, 835–849.e21. 10.1016/j.cell.2019.06.024 31327527 PMC6703186

[B140] Negm L. Chung J. Nobre L. Bennett J. Fernandez N. R. Nunes N. M. et al. (2025). The landscape of primary mismatch repair deficient gliomas in children, adolescents, and young adults: A multi-cohort study. *Lancet Oncol.* 26, 123–135. 10.1016/S1470-2045(24)00640-5 39701117

[B141] Nejo T. Krishna S. Yamamichi A. Lakshmanachetty S. Jimenez C. Lee K. Y. et al. (2025). Glioma-neuronal circuit remodeling induces regional immunosuppression. *Nat. Commun.* 16:4770. 10.1038/s41467-025-60074-z 40404658 PMC12098748

[B142] Nissen I. Dakhel S. Chakraborty C. Nygaard A. H. Kirkeby A. Hörnblad A. et al. (2025). Gene regulatory networks linked to GABA signalling emerge as relevant for glioblastoma pathogenesis. *bioRxiv* [Preprint] 10.1101/2025.04.30.651564

[B143] Nomura M. Spitzer A. Johnson K. C. Garofano L. Nehar-belaid D. Galili Darnell N. et al. (2025). The multilayered transcriptional architecture of glioblastoma ecosystems. *Nat. Genet.* 57, 1155–1167. 10.1038/s41588-025-02167-5 40346361 PMC12081307

[B144] Notarangelo G. Spinelli J. B. Perez E. M. Baker G. J. Kurmi K. Elia I. (2022). Oncometabolite d-2HG alters T cell metabolism to impair CD8T cell function. *Science* 377, 1519–1529. 10.1126/science.abj5104 36173860 PMC9629749

[B145] Ohtaka-Maruyama C. Okamoto M. Toyoda K. Yamaguchi A. Kobayashi N. Honda A. (2018). Synaptic transmission from subplate neurons controls radial migration of neocortical neurons. *Science* 360, 313–317. 10.1126/science.aar2866 29674592

[B146] Oldenborg P. A. Zheleznyak A. Fang Y. F. Lagenaur C. F. Gresham H. D. Lindberg F. P. (2000). Role of CD47 as a marker of self on red blood cells. *Science* 288, 2051–2054. 10.1126/science.288.5473.2051 10856220

[B147] Olson M. L. Mause E. R. V. Radhakrishnan S. V. Brody J. D. Rapoport A. P. Welm A. L. et al. (2022). Low-affinity CAR T cells exhibit reduced trogocytosis, preventing rapid antigen loss, and increasing CAR T cell expansion. *Leukemia* 36, 1943–1946. 10.1038/s41375-022-01585-2 35490197 PMC9252916

[B148] Ortiz B. Fabius A. W. M. Wu W. H. Pedraza A. Brennan C. W. Schultz N. et al. (2014). Loss of the tyrosine phosphatase PTPRD leads to aberrant STAT3 activation and promotes gliomagenesis. *Proc. Natl. Acad. Sci. U. S A.* 111, 8149–8154. 10.1073/pnas.1401952111 24843164 PMC4050622

[B149] Osswald M. Jung E. Sahm F. (2015). Brain tumour cells interconnect to a functional and resistant network. *Nature* 528, 93–98. 10.1038/nature16071 26536111

[B150] Ostrom Q. T. Patil N. Cioffi G. Waite K. Kruchko C. Barnholtz-Sloan J. S. (2020). CBTRUS statistical report: Primary brain and other central nervous system tumors diagnosed in the United States in 2013-2017. *Neuro Oncol.* 22(12 Suppl. 2), iv1–iv96. 10.1093/neuonc/noaa200 33123732 PMC7596247

[B151] Ostrom Q. T. Price M. Neff C. Cioffi G. Waite K. A. Kruchko C. et al. (2024). CBTRUS statistical report: Primary brain and other central nervous system tumors diagnosed in the United States in 2017-2021. *Neuro Oncol.* 26(Suppl. 6), vi1–vi85. 10.1093/neuonc/noae145 39371035 PMC11456825

[B152] Paez-Gonzalez P. Asrican B. Rodriguez E. Kuo C. T. (2014). Identification of distinct ChAT+ neurons and activity-dependent control of postnatal SVZ neurogenesis. *Nat. Neurosci.* 17, 934–942. 10.1038/nn.3734 24880216 PMC4122286

[B153] Park H. Poo M. M. (2013). Neurotrophin regulation of neural circuit development and function. *Nat. Rev. Neurosci.* 14, 7–23. 10.1038/nrn3379 23254191

[B154] Patel A. P. Tirosh I. Trombetta J. J. Shalek A. K. Gillespie S. M. Wakimoto H. (2014). Single-cell RNA-seq highlights intratumoral heterogeneity in primary glioblastoma. *Science* 344, 1396–1401. 10.1126/science.1254257 24925914 PMC4123637

[B155] Piao Y. Henry V. Tiao N. Park S. Y. Martinez-Ledesma J. Dong J. W. et al. (2017). Targeting intercellular adhesion molecule-1 prolongs survival in mice bearing bevacizumab-resistant glioblastoma. *Oncotarget* 8, 96970–96983. 10.18632/oncotarget.18859 29228586 PMC5722538

[B156] Pombo Antunes A. R. Scheyltjens I. Lodi F. (2021). Single-cell profiling of myeloid cells in glioblastoma across species and disease stage reveals macrophage competition and specialization. *Nat. Neurosci.* 24, 595–610. 10.1038/s41593-020-00789-y 33782623

[B157] Priego N. Zhu L. Monteiro C. Mulders M. Wasilewski D. Bindeman W. et al. (2018). STAT3 labels a subpopulation of reactive astrocytes required for brain metastasis. *Nat. Med.* 24, 1024–1035. 10.1038/s41591-018-0044-4 29892069

[B158] Quail D. F. Joyce J. A. (2017). The microenvironmental landscape of brain tumors. *Cancer Cell* 31, 326–341. 10.1016/j.ccell.2017.02.009 28292436 PMC5424263

[B159] Reardon D. A. Brandes A. A. Omuro A. Mulholland P. Lim M. Wick A. (2020). Effect of nivolumab vs bevacizumab in patients with recurrent glioblastoma: The checkMate 143 phase 3 randomized clinical Trial. *JAMA Oncol.* 6, 1003–1010. 10.1001/jamaoncol.2020.1024 32437507 PMC7243167

[B160] Ribeiro F. C. P. Gonçalves M. W. A. Silva A. M. Maciel T. F. de Lima-Souza R. A. Scarini J. F. et al. (2025). Beyond cell-cell contact: Therapeutic potential of Eph signaling in central nervous system tumors. *Front. Mol. Neurosci.* 18:1658651. 10.3389/fnmol.2025.1658651 41200151 PMC12586081

[B161] Roskoski R. Jr. (2012). ERK1/2 MAP kinases: Structure, function, and regulation. *Pharmacol. Res.* 66, 105–143. 10.1016/j.phrs.2012.04.005 22569528

[B162] Rubin J. B. Kung A. L. Klein R. S. Chan J. A. Sun Y. Schmidt K. et al. (2003). A small-molecule antagonist of CXCR4 inhibits intracranial growth of primary brain tumors. *Proc. Natl. Acad. Sci U. S. A.* 100, 13513–13518. 10.1073/pnas.2235846100 14595012 PMC263845

[B163] Ruiz-Rodríguez V. M. Torres-González C. A. Salas-Canedo K. M. Pecina-Maza N. Q. Martínez-Leija M. E. Portales-Pérez D. P. et al. (2023). Dynamical changes in the expression of GABAergic and purinergic components occur during the polarization of THP-1 monocytes to proinflammatory macrophages. *Biochem. Biophys. Rep.* 36:101558. 10.1016/j.bbrep.2023.101558 37881409 PMC10594599

[B164] Sakamoto K. Ozaki T. Suzuki Y. Kadomatsu K. (2021). Type IIa RPTPs and glycans: Roles in axon regeneration and synaptogenesis. *Int. J. Mol. Sci.* 22:5524. 10.3390/ijms22115524 34073798 PMC8197235

[B165] Salmaggi A. Corno C. Maschio M. Donzelli S. D’Urso A. Perego P. et al. (2021). Synergistic effect of perampanel and temozolomide in human glioma cell lines. *J. Pers. Med.* 11:390. 10.3390/jpm11050390 34068749 PMC8150827

[B166] Sampson J. H. Gunn M. D. Fecci P. E. Ashley D. M. (2020). Brain immunology and immunotherapy in brain tumours. *Nat. Rev. Cancer* 20, 12–25. 10.1038/s41568-019-0224-7 31806885 PMC7327710

[B167] Scherer H. J. (1938). Structural development in gliomas. *Am. J. Cancer* 34, 333–351.

[B168] Shanshiashvili L. Tsitsilashvili E. Dabrundashvili N. Kalandadze I. Mikeladze D. (2017). Metabotropic glutamate receptor 5 may be involved in macrophage plasticity. *Biol. Res.* 50:4. 10.1186/s40659-017-0110-2 28196513 PMC5310073

[B169] Shatz C. J. (2009). MHC class I: An unexpected role in neuronal plasticity. *Neuron* 64, 40–45. 10.1016/j.neuron.2009.09.044 19840547 PMC2773547

[B170] Shyntar A. Hillen T. (2026). Calcium signalling in glioblastoma networks of different topologies and possible treatments. *Math. Biosci.* 394:109638. 10.1016/j.mbs.2026.109638 41654173

[B171] Sigal Y. M. Speer C. M. Babcock H. P. Zhuang X. (2015). Mapping synaptic input fields of neurons with super-resolution imaging. *Cell* 163, 493–505. 10.1016/j.cell.2015.08.033 26435106 PMC4733473

[B172] Sochal M. Ditmer M. Gabryelska A. Białasiewicz P. (2022). The role of brain-derived neurotrophic factor in immune-related diseases: A narrative review. *J. Clin. Med.* 11:6023. 10.3390/jcm11206023 36294343 PMC9604720

[B173] Sottoriva A. Spiteri I. Piccirillo S. G. M. Touloumis A. Collins V. P. Marioni J. C. (2013). Intratumor heterogeneity in human glioblastoma reflects cancer evolutionary dynamics. *Proc. Natl. Acad. Sci. U. S. A.* 110, 4009–4014. 10.1073/pnas.1219747110 23412337 PMC3593922

[B174] Spruston N. (2008). Pyramidal neurons: Dendritic structure and synaptic integration. *Nat. Rev. Neurosci.* 9, 206–221. 10.1038/nrn2286 18270515

[B175] Stellwagen D. Beattie E. C. Seo J. Y. Malenka R. C. (2005). Differential regulation of AMPA receptor and GABA receptor trafficking by tumor necrosis factor-alpha. *J. Neurosci.* 25, 3219–3228. 10.1523/jneurosci.4486-04.2005 15788779 PMC6725093

[B176] Stevens B. Allen N. J. Vazquez L. E. Howell G. R. Christopherson K. S. Nouri N. (2007). The classical complement cascade mediates CNS synapse elimination. *Cell* 131, 1164–1178. 10.1016/j.cell.2007.10.036 18083105

[B177] Stinchcombe J. C. Majorovits E. Bossi G. Fuller S. Griffiths G. M. (2006). Centrosome polarization delivers secretory granules to the immunological synapse. *Nature* 443, 462–465. 10.1038/nature05071 17006514

[B178] Stoll S. Delon J. Brotz T. M. Germain R. N. (2002). Dynamic imaging of T cell-dendritic cell interactions in lymph nodes. *Science* 296, 1873–1876. 10.1126/science.1071065 12052961

[B179] Strogatz S. H. (2001). Exploring complex networks. *Nature* 410, 268–276. 10.1038/35065725 11258382

[B180] Stuart G. J. Spruston N. (2015). Dendritic integration: 60 years of progress. *Nat. Neurosci.* 18, 1713–1721. 10.1038/nn.4157 26605882

[B181] Stupp R. Hegi M. E. Gorlia T. Erridge S. C. Perry J. Hong Y. K. et al. (2014). Cilengitide combined with standard treatment for patients with newly diagnosed glioblastoma with methylated MGMT promoter (CENTRIC EORTC 26071-22072 study): A multicentre, randomised, open-label, phase 3 trial. *Lancet Oncol.* 15, 1100–1108. 10.1016/s1470-2045(14)70379-1 25163906

[B182] Stupp R. Mason W. P. Bent M. J. V. D. Weller M. Fisher B. Taphoorn M. J. B. et al. (2005). Radiotherapy plus concomitant and adjuvant temozolomide for glioblastoma. *New Engl. J. Med.* 352, 987–996. 10.1056/NEJMoa043330 15758009

[B183] Stupp R. Taillibert S. Kanner A. (2017). Effect of tumor-treating fields plus maintenance temozolomide vs maintenance temozolomide alone on survival in patients with glioblastoma: A randomized clinical trial. *JAMA* 318, 2306–2316. 10.1001/jama.2017.18718 29260225 PMC5820703

[B184] Südhof T. C. (2017). Synaptic neurexin complexes: A molecular code for the logic of neural circuits. *Cell* 171, 745–769. 10.1016/j.cell.2017.10.024 29100073 PMC5694349

[B185] Sun F. Zhou J. Dai B. Qian T. Zeng J. Li X. et al. (2020). Next-generation GRAB sensors for monitoring dopaminergic activity in vivo. *Nat. Methods* 17, 1156–1166. 10.1038/s41592-020-00981-9 33087905 PMC7648260

[B186] Sun Y. Wang X. Zhang D. Y. Zhang Z. Bhattarai J. P. Wang Y. et al. (2025a). Brain-wide neuronal circuit connectome of human glioblastoma. *Nature* 641, 222–231. 10.1038/s41586-025-08634-7 39821165 PMC12347542

[B187] Sun Y. Wang X. Zhang Z. Park K. H. Wu Y. Dong W. et al. (2025b). Cholinergic neuron-to-glioblastoma synapses in a human iPSC-derived co-culture model. *Stem Cell Rep.* 20:102534. 10.1016/j.stemcr.2025.102534 40541171 PMC12277791

[B188] Sweatt J. D. (2004). Mitogen-activated protein kinases in synaptic plasticity and memory. *Curr. Opin. Neurobiol.* 14, 311–317. 10.1016/j.conb.2004.04.001 15194111

[B189] Takahashi H. Craig A. M. (2013). Protein tyrosine phosphatases PTPδ, PTPσ, and LAR: Presynaptic hubs for synapse organization. *Trends Neurosci.* 36, 522–534. 10.1016/j.tins.2013.06.002 23835198 PMC3789601

[B190] Tarassishin L. Casper D. Lee S. C. (2014). Aberrant expression of interleukin-1β and inflammasome activation in human malignant gliomas. *PLoS One* 9:e103432. 10.1371/journal.pone.0103432 25054228 PMC4108401

[B191] Taylor K. R. Barron T. Hui A. Spitzer A. Yalçin B. Ivec A. E. (2023). Glioma synapses recruit mechanisms of adaptive plasticity. *Nature* 623, 366–374. 10.1038/s41586-023-06678-1 37914930 PMC10632140

[B192] Tetzlaff S. K. Reyhan E. Layer N. Bengtson C. P. Heuer A. Schroers J. (2025). Characterizing and targeting glioblastoma neuron-tumor networks with retrograde tracing. *Cell* 188, 390–411.e36. 10.1016/j.cell.2024.11.002 39644898

[B193] Tomaszewski W. H. Waibl-Polania J. Chakraborty M. Perera J. Ratiu J. Miggelbrink A. et al. (2022). Neuronal CaMKK2 promotes immunosuppression and checkpoint blockade resistance in glioblastoma. *Nat. Commun.* 13:6483. 10.1038/s41467-022-34175-y 36309495 PMC9617949

[B194] Tralongo P. Ballato M. Zuccala V. Fiorentino V. Giordano W. Casili G. et al. (2026). Neurotransmitter-Mediated signaling in glioblastoma and glial tumors: Biology and therapeutic opportunities. *Oncol. Res.* 34:1. 10.32604/or.2026.076088 42220436 PMC13220060

[B195] Veeriah S. Brennan C. Meng S. Singh B. Fagin J. A. Solit D. B. et al. (2009). The tyrosine phosphatase PTPRD is a tumor suppressor that is frequently inactivated and mutated in glioblastoma and other human cancers. *Proc. Natl. Acad. Sci. U. S. A.* 106, 9435–9440. 10.1073/pnas.0900571106 19478061 PMC2687998

[B196] Venkataramani V. Tanev D. I. Strahle C. (2019). Glutamatergic synaptic input to glioma cells drives brain tumour progression. *Nature* 573, 532–538. 10.1038/s41586-019-1564-x 31534219

[B197] Venkataramani V. Yang Y. Schubert M. C. (2022). Glioblastoma hijacks neuronal mechanisms for brain invasion. *Cell* 185, 2899–2917.e31. 10.1016/j.cell.2022.06.054 35914528

[B198] Venkatesh H. S. Johung T. B. Caretti V. (2015). Neuronal activity promotes glioma growth through neuroligin-3 secretion. *Cell* 161, 803–816. 10.1016/j.cell.2015.04.012 25913192 PMC4447122

[B199] Venkatesh H. S. Morishita W. Geraghty A. C. (2019). Electrical and synaptic integration of glioma into neural circuits. *Nature* 573, 539–545. 10.1038/s41586-019-1563-y 31534222 PMC7038898

[B200] Venkatesh H. S. Tam L. T. Woo P. J. Lennon J. Nagaraja S. Gillespie S. M. et al. (2017). Targeting neuronal activity-regulated neuroligin-3 dependency in high-grade glioma. *Nature* 549, 533–537. 10.1038/nature24014 28959975 PMC5891832

[B201] Wang L. Jung J. Babikir H. Shamardani K. Jain S. Feng X. et al. (2022). A single-cell atlas of glioblastoma evolution under therapy reveals cell-intrinsic and cell-extrinsic therapeutic targets. *Nat. Cancer* 3, 1534–1552. 10.1038/s43018-022-00475-x 36539501 PMC9767870

[B202] Wang Y. Wang Y. Bao H. Wang Z. Jiang T. (2025). Mechanisms and therapeutic implications of glioma-neuron interactions. *Cancer Lett.* 629:217884. 10.1016/j.canlet.2025.217884 40545026

[B203] Watson D. C. Bayik D. Storevik S. Moreino S. S. Sprowls S. A. Han J. et al. (2023). GAP43-dependent mitochondria transfer from astrocytes enhances glioblastoma tumorigenicity. *Nat. Cancer* 4, 648–664. 10.1038/s43018-023-00556-5 37169842 PMC10212766

[B204] Wayman G. A. Lee Y. S. Tokumitsu H. Silva A. J. Soderling T. R. (2008). Calmodulin-kinases: Modulators of neuronal development and plasticity. *Neuron* 59, 914–931. 10.1016/j.neuron.2008.08.021 18817731 PMC2664743

[B205] Weil S. Osswald M. Solecki G. Grosch J. Jung E. Lemke D. et al. (2017). Tumor microtubes convey resistance to surgical lesions and chemotherapy in gliomas. *Neuro Oncol.* 19, 1316–1326. 10.1093/neuonc/nox070 28419303 PMC5596180

[B206] Weller M. van den Bent M. Preusser M. Le Rhun E. Tonn J. C. Minniti G. et al. (2021). EANO guidelines on the diagnosis and treatment of diffuse gliomas of adulthood. *Nat. Rev. Clin. Oncol.* 18, 170–186. 10.1038/s41571-020-00447-z 33293629 PMC7904519

[B207] Wen P. Y. Kesari S. (2008). Malignant gliomas in adults. *N. Engl. J. Med.* 359, 492–507. 10.1056/NEJMra0708126 18669428

[B208] West A. E. Greenberg M. E. (2011). Neuronal activity-regulated gene transcription in synapse development and cognitive function. *Cold Spring Harb. Perspect. Biol.* 3:a005744. 10.1101/cshperspect.a005744 21555405 PMC3098681

[B209] Wheeler M. A. Quintana F. J. (2025). The neuroimmune connectome. *Nature* 638, 333–342. 10.1038/s41586-024-08474-x 39939792 PMC12039074

[B210] Whipple N. Molinaro A. Reddy A. Jaeger-Krause M. Iv M. Villanueva-Meyer J. et al. (2025). CTP-18. PNOC025: A phase 1 study assessing the safety and tolerability of magrolimab in children and adults with recurrent or progressive malignant brain tumors. *Neuro Oncol.* 27(Suppl._5), v149–v150. 10.1093/neuonc/noaf201.0590

[B211] Wirsching H. G. Roth P. Weller M. (2021). A vasculature-centric approach to developing novel treatment options for glioblastoma. *Expert Opin. Ther. Targets* 25, 87–100. 10.1080/14728222.2021.1881062 33482697

[B212] Woroniecka K. Chongsathidkiet P. Rhodin K. Kemeny H. Dechant C. Farber S. H. et al. (2018). T-Cell exhaustion signatures vary with tumor type and are severe in glioblastoma. *Clin. Cancer Res.* 24, 4175–4186. 10.1158/1078-0432.CCR-17-1846 29437767 PMC6081269

[B213] Wu A. Maxwell R. Xia Y. Cardarelli P. Oyasu M. Belcaid Z. et al. (2019). Combination anti-CXCR4 and anti-PD-1 immunotherapy provides survival benefit in glioblastoma through immune cell modulation of tumor microenvironment. *J. Neuro-Oncol.* 143, 241–249. 10.1007/s11060-019-03172-5 31025274

[B214] Wu Y. Hu Y. Tang L. Yin S. Lv L. Zhou P. (2022). Targeting CXCR4 to suppress glioma-initiating cells and chemoresistance in glioma. *Cell Biol. Intern.* 46, 1519–1529. 10.1002/cbin.11836 35731168

[B215] Yadav N. Purow B. W. (2024). Understanding current experimental models of glioblastoma-brain microenvironment interactions. *J. Neurooncol.* 166, 213–229. 10.1007/s11060-023-04536-8 38180686 PMC11056965

[B216] Yang G. Niu X. Gan J. Yu T. Li M. Xiao P. et al. (2025). Glioblastoma exploits ATP from leading-edge astrocytes to fuel its infiltrative growth revealed by spatially resolved chimeric analysis. *Sci. Adv.* 11:eadv5673. 10.1126/sciadv.adv5673 40991701 PMC12459425

[B217] Yang X. Annaert W. (2021). The nanoscopic organization of synapse structures: A common basis for cell communication. *Membranes* 11:248. 10.3390/membranes11040248 33808285 PMC8065904

[B218] Ye Z. C. Sontheimer H. (1999). Glioma cells release excitotoxic concentrations of glutamate. *Cancer Res.* 59, 4383–4391. 10485487

[B219] Yokosuka T. Takamatsu M. Kobayashi-Imanishi W. Hashimoto-Tane A. Azuma M. Saito T. (2012). Programmed cell death 1 forms negative costimulatory microclusters that directly inhibit T cell receptor signaling by recruiting phosphatase SHP2. *J. Exp. Med.* 209, 1201–1217. 10.1084/jem.20112741 22641383 PMC3371732

[B220] Yoo K. C. Kang J. H. Choi M. Y. Suh Y. Zhao Y. Kim M. J. et al. (2022). Soluble ICAM-1 a pivotal communicator between tumors and macrophages, promotes mesenchymal shift of glioblastoma. *Adv. Sci.* 9:e2102768. 10.1002/advs.202102768 34813169 PMC8805565

[B221] Yoshida Y. (2012). Semaphorin signaling in vertebrate neural circuit assembly. *Front. Mol. Neurosci.* 5:71. 10.3389/fnmol.2012.00071 22685427 PMC3368236

[B222] Yu H. Lee H. Herrmann A. Buettner R. Jove R. (2014). Revisiting STAT3 signalling in cancer: New and unexpected biological functions. *Nat. Rev. Cancer* 14, 736–746. 10.1038/nrc3818 25342631

[B223] Zhang W. Wang J. Ji J. Wang P. Yuan G. Fang S. et al. (2025). Glioblastoma cells secrete ICAM1 via FASN signaling to promote glioma-associated macrophage infiltration. *Cell. Signal.* 132:111823. 10.1016/j.cellsig.2025.111823 40252818

[B224] Zhang X. Xue J. Zhao C. Zeng C. Zhong J. Yu G. et al. (2026). Oligodendrocyte transcription factor 2 orchestrates glioblastoma immune evasion by suppressing CXCL10 and CD8+ T cell activation. *J. Clin. Invest.* 136:e195556. 10.1172/jci195556 41591814 PMC12948422

[B225] Zhang Y. Kang T. Wang Y. Song C. Li H. Mi H. et al. (2024). A low level of tumor necrosis factor α in tumor microenvironment maintains the self-renewal of glioma stem cells by Vasorin-mediated glycolysis. *Neuro Oncol.* 26, 2256–2271. 10.1093/neuonc/noae147 39093693 PMC11630517

[B226] Zhao G. Gentile M. E. Xue L. Cosgriff C. V. Weiner A. I. Adams-Tzivelekidis S. et al. (2024). Vascular endothelial-derived SPARCL1 exacerbates viral pneumonia through pro-inflammatory macrophage activation. *Nat. Commun.* 15:4235. 10.1038/s41467-024-48589-3 38762489 PMC11102455

[B227] Zhao J. Bang S. Furutani K. McGinnis A. Jiang C. Roberts A. et al. (2023). PD-L1/PD-1 checkpoint pathway regulates hippocampal neuronal excitability and learning and memory behavior. *Neuron* 111, 2709–2726.e9. 10.1016/j.neuron.2023.05.022 37348508 PMC10529885

